# Joint Association of Physical Activity and Prognostic Nutritional Index on Survival in US Cancer Survivors: A Study Based on the NHANES Database

**DOI:** 10.1002/cam4.71767

**Published:** 2026-05-10

**Authors:** Linli Chen, Yinhao Chen, Yutao Li, Yuhan Li, Xiang Ruan, Paula Tups, Ingo G. H. Schmidt‐Wolf

**Affiliations:** ^1^ Department of Integrated Oncology, Center for Integrated Oncology (CIO) University Hospital Bonn Bonn Germany

**Keywords:** cancer survivors, exercise–nutrition interaction, mortality risk, physical activity, Prognostic Nutritional Index

## Abstract

**Background:**

Nutritional–immune status and physical activity (PA) are key to prognosis in cancer survivors, yet their joint association with mortality remains unclear. Therefore, we examined the independent and combined effects of PA and the Prognostic Nutritional Index (PNI) on mortality outcomes.

**Methods:**

We evaluated the independent and joint associations of PA and PNI with all‐cause, cancer‐specific, non‐cancer, and cardiovascular mortality among 2420 US cancer survivors using data from the 2007–2016 National Health and Nutrition Examination Survey (NHANES). Prognostic Nutritional Index was used as an indicator of nutritional–immune status. We used multivariable Cox regression, extended Cox regression models, and restricted cubic spline analyses to evaluate the associations and dose–response relationships. Combined associations were examined using joint PNI–PA exposure models and Kaplan–Meier curves; multiplicative interaction was tested with likelihood ratio tests, and additive interaction was quantified using relative excess risk due to interaction (RERI), attributable proportion (AP), and synergy index (SI). Sensitivity analyses were performed using median and tertile PNI cut‐off points. Meanwhile, subgroup analyses were conducted to investigate heterogeneity.

**Results:**

Prognostic Nutritional Index was overall inversely associated with mortality risk (all‐cause: HR = 0.762, cancer‐specific: HR = 0.741, non‐cancer: HR = 0.771, cardiovascular mortality: HR = 0.798; all *p* < 0.001), but this protective association was not constant and gradually attenuated over follow‐up time. There was a graded inverse association between PA and mortality. Joint analyses revealed that the group with high PNI and sufficient PA was linked to an approximately 70% lower risk of all‐cause mortality (*p* < 0.01). No multiplicative interaction was detected, whereas additive interaction was observed for all‐cause mortality (RERI = 0.52; AP = 0.23; SI = 1.69). Sensitivity analyses supported the practical utility of PNI categorization, and subgroup analyses highlighted potentially heterogeneous associations across cancer types.

**Conclusion:**

Prognostic Nutritional Index and PA were independently and jointly associated with survival among cancer survivors, supporting integrated survivorship strategies targeting nutritional–immune status and physical activity.

## 1 Introduction

Cancer has emerged as a significant global public health concern, with its incidence and mortality rates steadily rising in recent years. According to the latest statistics, global cancer incidence reached 20 million in 2022, and this figure is expected to reach 35 million by 2050 [1]. Although advancements in medical technology have significantly improved long‐term survival rates among cancer patients [2], research indicates that many survivors continue to experience various adverse health outcomes during rehabilitation, including cardiovascular complications, metabolic abnormalities, and psychological disorders [3]. Consequently, developing effective and practical intervention strategies to improve the quality of life and long‐term prognosis of cancer survivors has become a critical focus of both clinical practice and public health research.

Physical activity (PA), as a crucial modifiable factor, plays a significant role in reducing disease risk and minimizing complications by enhancing cardiovascular function, improving metabolism, and strengthening the immune response [4]. Research has demonstrated that PA can lower the risk of at least 13 types of cancer [5] and serves as an effective adjunct in mitigating sequelae during cancer treatment [6]. Multiple cohort studies and guidelines from leading academic institutions, such as the American College of Sports Medicine (ACSM) and the American Cancer Society (ACS) [7] have consistently demonstrated that regular moderate‐intensity exercise can effectively reduce the risk of cancer recurrence and improve survival rates. These benefits are mediated through mechanisms including enhanced cardiovascular function, reduced chronic inflammation, metabolic regulation, and improved insulin sensitivity [8].

Meanwhile, nutritional status is a pivotal determinant in the recovery and long‐term outcomes of cancer patients, particularly in mitigating the side effects of treatment and supporting immune function [9, 10]. The Prognostic Nutritional Index (PNI) is a valuable tool for assessing prognosis and predicting survival rates, as it is a comprehensive indicator of nutritional and immune status that integrates serum albumin levels and lymphocyte count [11]. Studies have shown that higher PNI levels are linked to a lower incidence of surgical complications, better responses to treatment, and higher survival rates. Conversely, lower PNI levels are often an indicator of a poorer prognosis [12].

Although the individual effects of PA and the PNI on cancer prognosis are relatively well understood, research on their combined impact remains limited. Most studies have focused on specific cancer types or small sample populations [13]. To address this issue, the present study systematically evaluates the independent and interactive effects of PA and PNI on the survival of US cancer survivors using data from the nationally representative National Health and Nutrition Examination Survey (NHANES). In the study, we provide population‐level evidence of the associations between PA and nutritional–immune status, both independently and jointly, with mortality among cancer survivors.

## 2 Methods

### 2.1 Data Source

This study used an observational cohort analysis of NHANES data and was reported in accordance with the STROBE guidelines. NHANES is an ongoing, nationally representative cross‐sectional survey initiated by the Centers for Disease Control and Prevention (CDC) to assess the health and nutritional status of non‐institutionalized US residents. For this study, we analyzed six data cycles from 2007 to 2016, focusing on individuals with a confirmed cancer diagnosis. Data on health behaviors, nutritional status, and follow‐up outcomes were collected in order to systematically examine the independent and combined effects of PA and the PNI on cancer survivors' survival outcomes.

### 2.2 Study Subjects

**FIGURE 1:**
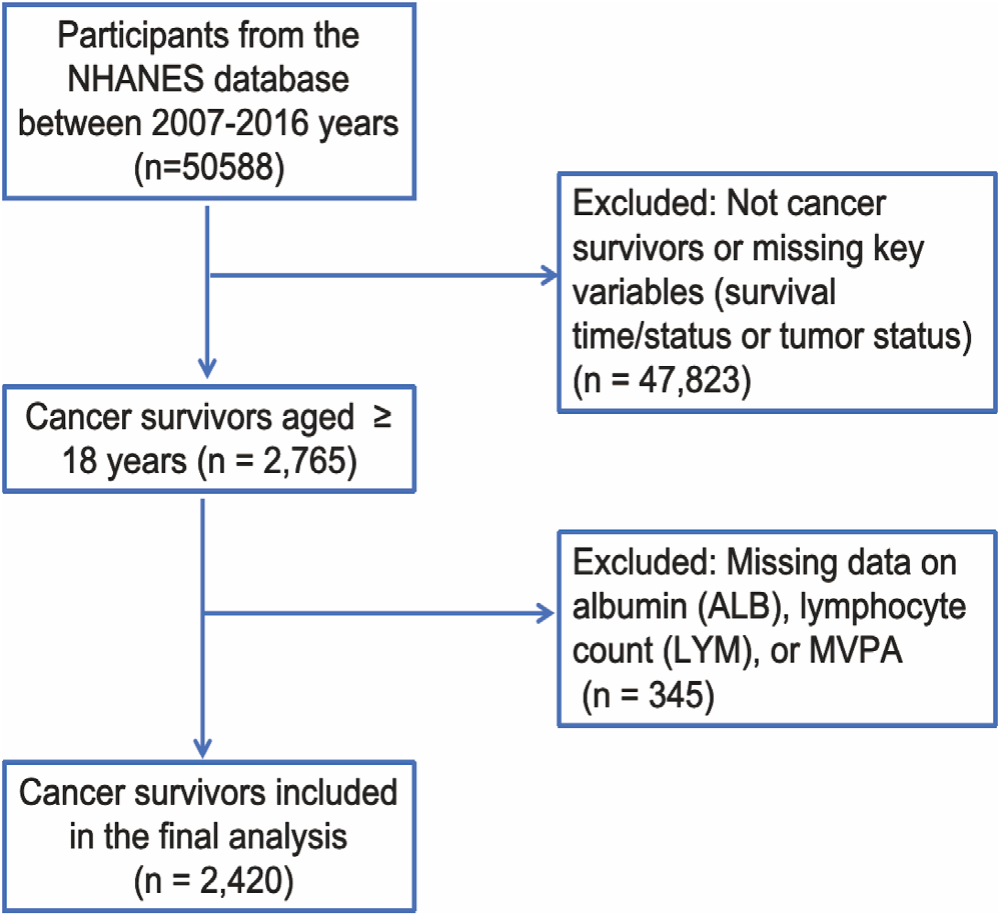
Study cohort selection flowchart.

Inclusion Criteria: (1) Individuals who have been diagnosed with cancer by medical professionals, based on the following survey question: “Has a doctor ever informed you that you have any type of cancer or malignant tumor?” (2) Participants must be aged 18 years or over. (3) Participants must have complete data on PA, nutritional status, and follow‐up outcomes. Exclusion criteria: (1) individuals with missing key demographic or follow‐up information; (2) participants with an unclear cancer diagnosis; (3) participants with missing survival information. A total of 2420 cancer survivors who met the inclusion criteria were included in the study. The sample selection process is illustrated in Figure 1.

### 2.3 Physical Activity

The total duration of PA was reported by participants through self‐assessment using the Global Physical Activity Questionnaire (GPAQ), an instrument developed by the World Health Organization (WHO) to evaluate PA across multiple [14], including occupational activities, transportation methods and leisure‐time activities. A detailed GPAQ analysis guide is available at: WHO GPAQ Analysis Guide. According to WHO analysis guidelines, each participant's PA data was converted into weekly Metabolic Equivalent Task (MET) minutes in order to quantify their moderate‐to‐vigorous PA level. The MET value depends on the type of activity performed, with reference values established by NHANES for different PA categories. The PA score was computed using the equation: MET‐min/week = MET value × weekly frequency (days) × duration per activity (minutes). According to the US Physical Activity Guidelines [15], adults are advised to accumulate either 150 min of moderate‐intensity PA or 75 min of vigorous‐intensity PA weekly, corresponding to 600 MET‐min/week. Following these recommendations, PA levels in this study were classified into three categories: (1) inactive: 0 MET‐min/week, no moderate or vigorous activity; (2) insufficiently active: 1–599 MET‐min/week, below the minimum recommended standard; (3) sufficiently active: ≥ 600 MET‐min/week, meeting or exceeding the minimum recommended standard.

### 2.4 Prognostic Nutritional Index

In this study, the PNI was calculated using the formula: 5 × lymphocyte count (10^9^/L) + serum albumin (g/L) [16]. Serum albumin and lymphocyte counts were obtained from the NHANES laboratory component and measured according to standardized protocols with quality‐control procedures implemented by the National Center for Health Statistics. Lymphocyte count was derived from complete blood count (CBC) testing using hematology methods based on Beckman Coulter technology [17], and serum albumin was quantified using the bromocresol green method on the Roche Cobas 6000 analyzer (Roche Diagnostics, Indianapolis, IN). PNI was primarily modeled as a continuous variable in the main analyses to preserve information, and its dose–response relationship with mortality was further evaluated using restricted cubic spline (RCS) analyses based on the fully adjusted model. To improve interpretability and enable joint analyses with PA, participants were additionally categorized by the median PNI value in the analytic sample into low PNI (≤ 51) and high PNI (> 51) groups. In sensitivity analyses, PNI was alternatively categorized into tertiles to assess the robustness of the findings. Among available nutritional indices, PNI was selected because it provides a parsimonious, objective, and reproducible measure of nutritional–immune status based on standardized NHANES laboratory data (serum albumin and lymphocyte count), making it well suited to population‐based analyses and joint modeling with PA.

### 2.5 Outcome Variables

The outcome variables in this study encompass multiple dimensions to comprehensively evaluate the influence of PA and PNI on survival outcomes among cancer survivors. The primary outcome was all‐cause mortality, which was defined as death from any cause during the follow‐up period. The secondary outcomes were cancer‐specific mortality, non‐cancer mortality, and cardiovascular disease (CVD) mortality. The cause of death was determined by linking NHANES participants to the National Death Index (NDI), with the underlying cause classified according to the International Classification of Diseases, 10th Revision (ICD‐10). Cancer‐specific mortality was defined as deaths attributed to malignant neoplasms, whereas non‐cancer mortality comprised deaths from causes other than cancer. CVD mortality was defined as deaths attributed to cardiovascular causes based on the ICD‐10‐coded underlying cause of death. Survival time was calculated in months from the date of the baseline NHANES examination to the date of death or the end of follow‐up (December 31, 2019). Participants who were alive at the end of the follow‐up period were censored on that date.

### 2.6 Covariates

Covariates were selected based on prior studies on lifestyle factors and cancer survivor prognosis [12, 18]. The demographic variables considered were age, sex, race, marital status, education, and poverty‐income ratio (PIR; non‐poor: PIR ≥ 1; poor: PIR < 1). Lifestyle factors were self‐reported and included smoking status, alcohol consumption, and PA levels. Alcohol consumption was determined using two 24‐h dietary recalls; participants who reported consuming alcohol in either recall were classified as drinkers. Smoking status was categorized as never (< 100 cigarettes), former (≥ 100 cigarettes and not currently smoking), or current (≥ 100 cigarettes and currently smoking daily or intermittently). PA was assessed using reported vigorous and moderate activities.

Anthropometric and metabolic measures, including body mass index (BMI), waist circumference, blood pressure, lipid profile, and fasting glucose were obtained from NHANES examinations. Obesity was defined as a BMI ≥ 30 kg/m^2^, and abdominal obesity was defined as a waist circumference > 88 cm in women and > 102 cm in men [19]. Hypertension diagnosis required either systolic/diastolic pressures ≥ 140/90 mmHg or a physician‐confirmed history [20]. Diabetes was defined as a physician diagnosis, fasting glucose ≥ 7.0 mmol/L, HbA1c > 6.5%, random glucose ≥ 11.1 mmol/L, 2‐h OGTT glucose ≥ 11.1 mmol/L, or use of hypoglycemic medications [21]. Hyperlipidemia was defined as total cholesterol ≥ 240 mg/dL, LDL‐C ≥ 160 mg/dL, HDL‐C < 40 mg/dL (males)/< 50 mg/dL (females), triglycerides ≥ 150 mg/dL, or lipid‐modifying therapy [22].

Cancer subtype was based on self‐reported primary cancer from the NHANES medical conditions questionnaire and was grouped into the three most prevalent cancer types in the analytic sample, with all others classified as “other.” According to the CDC definition of obesity‐associated cancers, cancers were further classified as obesity‐associated versus non‐obesity‐associated. The former includes adenocarcinoma of the esophagus, as well as cancers of the breast, colon and rectum, uterus, gallbladder, upper stomach, kidney, liver, ovary, pancreas, thyroid, meningioma, and multiple myeloma. All other cancer types were categorized as non‐obesity‐associated [23]. All data elements were systematically retrieved from standardized NHANES survey instruments and clinical examination protocols.

### 2.7 Statistical Analysis

The statistical procedures implemented in this investigation complied with NHANES analytical protocols, utilizing cluster sampling and stratification techniques to enhance the precision of variance estimation. Baseline demographic profiles were stratified according to PA classifications. Continuous variables with normal distributions were expressed as the mean ± standard deviation (SD) and analyzed via independent *t*‐tests, while categorical variables were presented as counts (percentages) and evaluated using Pearson's chi‐square test. Missing values in covariates were handled using multiple imputation by chained equations (MICE). The associations of PNI and PA with all‐cause, cancer‐specific, non‐cancer, and cardiovascular mortality were initially examined using multivariable Cox proportional hazards models. PNI was primarily modeled as a continuous variable and was also dichotomized at the median for joint analyses and to assess additive interaction. Three models were fitted: Model 1 was unadjusted; Model 2 was adjusted for age, sex, race, marital status, educational attainment and PIR; Model 3 was further adjusted for smoking status, alcohol consumption, BMI, hypertension, hyperlipidemia, diabetes, obesity, abdominal obesity, and cancer subtype. The potential nonlinearity of the PNI‐mortality association was evaluated using RCS based on the fully adjusted model framework. The proportional hazards assumption was assessed using Schoenfeld residual–based tests. When violation of the proportional hazards assumption was detected in the main models, extended Cox models with time‐varying coefficients were fitted by including covariate‐by‐log(time) interaction terms.

The interaction between PNI and PA was assessed on both multiplicative and additive scales. Multiplicative interaction was tested by adding a PNI × PA cross‐product term to Model 3 and evaluating it using a likelihood ratio test. Additive interaction was quantified using the relative excess risk due to interaction (RERI), AP, and synergy index (SI) based on a 2 × 2 joint exposure framework (low vs. high PNI; inactive vs. active PA, where active combines insufficiently and sufficiently active). Bootstrap resampling (1000 iterations) was used to derive 95% confidence intervals for additive interaction measures. Subgroup analyses were performed to explore heterogeneity by sex, socioeconomic status, metabolic conditions, cancer subtype, and cancer categories. Sensitivity analyses involved repeating Model 3 with PNI specified as either a median‐based binary variable or in tertiles.

All computational workflows were executed in the R statistical environment (version 4.2.3), with a significance threshold of *p* < 0.05 set for two‐tailed hypothesis testing.

## 3 Results

### 3.1 Baseline Characteristics Description

Of the 2420 cancer survivors included in the study, the median age was 65.14 years. Of these participants, 47.64% (*n* = 1153) were male and 52.36% (*n* = 1267) were female. The majority of participants were of non‐Hispanic White ethnicity (68.84%, *n* = 1666), and 56.28% (*n* = 1362) had a college degree or higher qualification. In addition, 60.58% (*n* = 1466) were married, and 85.66% (*n* = 2073) had a family poverty‐income ratio above the poverty level. With regard to lifestyle factors, 45.17% (*n* = 1093) had never smoked, 16.03% (*n* = 388) were current smokers, and 12.77% (*n* = 309) reported alcohol consumption. The prevalences of cardiometabolic comorbidities were as follows: hypertension (64.46%, *n* = 1560); hyperlipidemia (76.28%, *n* = 1846); diabetes (25.87%, *n* = 626); obesity (37.44%, *n* = 906); and abdominal obesity (66.74%, *n* = 1615).

Overall, the mean PNI was 51.57 (SD, 5.06) and differed between PA groups (*p* < 0.01). The distribution of cancer subtypes also varied by PA group (*p* < 0.01); non‐melanoma skin cancer (17.64%), breast cancer (15.08%), and prostate cancer (15.87%) were the most common, while other cancers accounted for 51.40%. Obesity‐related cancers accounted for 32.69% of the sample and differed across PA groups (*p* < 0.01). During a mean follow‐up period of 83.42 months (SD, 38.50), 26.86% of participants (*n* = 650) died, including 8.88% from cancer (*n* = 215), 6.90% from CVD (*n* = 167), and 17.98% from non‐cancer causes (*n* = 435). Collectively, these findings indicate that PA level is associated with multiple demographic and metabolic characteristics. Detailed characteristics stratified by PA level are presented in Table 1.

**TABLE 1:** The demographic characteristics of cancer survivors in the present study stratified by PA classification.

Variables	Overall	Inactive	Insufficiently active	Sufficiently active	*p*
	*N* = 2420	*N* = 1375	*N* = 771	*N* = 274	
**Age, mean (SD)**	65.14 (14.27)	66.55 (13.58)	63.74 (14.32)	62.03 (16.46)	< 0.001
**Sex, *n* (*p* %)**					
Male	1153 (47.64)	625 (45.45)	366 (47.47)	162 (59.12)	< 0.001
Female	1267 (52.36)	750 (54.55)	405 (52.53)	112 (40.88)	
**Race, *n* (%)**					
Mexican American	162 (6.69)	108 (7.85)	39 (5.06)	15 (5.47)	0.2
Non‐Hispanic Black	320 (13.22)	190 (13.82)	94 (12.19)	36 (13.14)	
Non‐Hispanic White	1666 (68.84)	922 (67.05)	553 (71.73)	191 (69.71)	
Other	272 (11.24)	155 (11.27)	85 (11.02)	32 (11.68)	
**Education, *n* (%)**					
<High school	527 (21.78)	404 (29.38)	92 (11.93)	31 (11.31)	< 0.001
> High school	1362 (56.28)	664 (48.29)	508 (65.89)	190 (69.34)	
Completed high school	531 (21.94)	307 (22.33)	171 (22.18)	53 (19.34)	
**Marital, *n* (%)**					
Married	1466 (60.58)	788 (57.31)	503 (65.24)	175 (63.87)	< 0.001
Other	954 (39.42)	587 (42.69)	268 (34.76)	99 (36.13)	
**PIR, *n* (%)**					
Not poor	2073 (85.66)	1137 (82.69)	687 (89.11)	249 (90.88)	< 0.001
Poor	347 (14.34)	238 (17.31)	84 (10.89)	25 (9.12)	
**Smoke, *n* (%)**					
Current	388 (16.03)	265 (19.27)	82 (10.64)	41 (14.96)	< 0.001
Former	939 (38.80)	521 (37.89)	308 (39.95)	110 (40.15)	
Never	1093 (45.17)	589 (42.84)	381 (49.42)	123 (44.89)	
**Alcohol, *n* (%)**					
No	2111 (87.23)	1249 (90.84)	648 (84.05)	214 (78.10)	< 0.001
Yes	309 (12.77)	126 (9.16)	123 (15.95)	60 (21.90)	
**BMI**	29.14 (6.56)	29.61 (6.71)	28.52 (6.54)	28.47 (5.53)	< 0.001
**Obesity, *n* (%)**					
No	1514 (62.56)	808 (58.76)	523 (67.83)	183 (66.79)	< 0.001
Yes	906 (37.44)	567 (41.24)	248 (32.17)	91 (33.21)	
**Abdominal obesity, *n* (%)**					
No	805 (33.26)	382 (27.78)	312 (40.47)	111 (40.51)	< 0.001
Yes	1615 (66.74)	993 (72.22)	459 (59.53)	163 (59.49)	
**Hypertension, *n* (%)**					
No	860 (35.54)	435 (31.64)	295 (38.26)	130 (47.45)	< 0.001
Yes	1560 (64.46)	940 (68.36)	476 (61.74)	144 (52.55)	
**Diabetes, *n* (%)**					
No	1794 (74.13)	957 (69.60)	619 (80.29)	218 (79.56)	< 0.001
Yes	626 (25.87)	418 (30.40)	152 (19.71)	56 (20.44)	
**Hyperlipidemia, *n* (%)**					
No	574 (23.72)	308 (22.40)	198 (25.68)	68 (24.82)	0.2
Yes	1846 (76.28)	1067 (77.60)	573 (74.32)	206 (75.18)	
**PNI, mean (SD)**	51.57 (5.06)	51.20 (5.22)	52.02 (4.84)	52.14 (4.71)	< 0.001
**Cancer subtype, *n* (%)**					
SkinNonMelanoma	427 (17.64)	213 (15.49)	162 (21.01)	52 (18.98)	0.003
Breast	365 (15.08)	222 (16.15)	117 (15.18)	26 (9.49)	
Prostate	384 (15.87)	212 (15.42)	121 (15.69)	51 (18.61)	
Other	1244 (51.40)	728 (52.95)	371 (48.12)	145 (52.92)	
**CancerCategory, *n* (%)**					
Non‐obesity‐related cancer	1629 (67.31)	872 (63.42)	559 (72.50)	198 (72.26)	< 0.001
Obesity‐related cancer	791 (32.69)	503 (36.58)	212 (27.50)	76 (27.74)	
**Follow‐up time, mean (SD)**	83.42 (38.50)	80.21 (39.62)	87.69 (36.75)	87.51 (36.12)	< 0.001
**All‐mortality, *n* (%)**					
No	1770 (73.14)	906 (65.89)	624 (80.93)	240 (87.59)	< 0.001
Yes	650 (26.86)	469 (34.11)	147 (19.07)	34 (12.41)	
**Cancer mortality, *n* (%)**					
No	2205 (91.12)	1224 (89.02)	720 (93.39)	261 (95.26)	< 0.001
Yes	215 (8.88)	151 (10.98)	51 (6.61)	13 (4.74)	
**Non‐cancer mortality, *n* (%)**					
No	1985 (82.02)	1057 (76.87)	675 (87.55)	253 (92.34)	< 0.001
Yes	435 (17.98)	318 (23.13)	96 (12.45)	21 (7.66)	
**CVD mortality, *n* (%)**					
No	2253 (93.10)	1258 (91.49)	729 (94.55)	266 (97.08)	< 0.001
Yes	167 (6.90)	117 (8.51)	42 (5.45)	8 (2.92)	

*Note:* inactive, 0 MET‐min/week; insufficiently active, 1–599 MET‐min/week; sufficiently active, ≥ 600 MET‐min/week. *p*‐value < 0.05 was considered significant.

Abbreviations: CVD mortality, cardiovascular disease mortality; PA, physical activity; PIR, poverty‐income ratio; PNI, Prognostic Nutritional Index.

### 3.2 Independent Effects of PA and PNI on Mortality

In the fully adjusted analyses, higher PA was consistently associated with lower mortality risk, and higher PNI remained inversely associated with mortality, although the association for PNI varied over follow‐up time in the extended Cox models. Specifically, the main effect of PNI was inversely associated with all‐cause mortality (HR = 0.762, 95% CI: 0.704–0.823; *p* < 0.001), cancer mortality (HR = 0.741, 95% CI: 0.655–0.839; *p* < 0.001), non‐cancer mortality (HR = 0.771, 95% CI: 0.697–0.853; *p* < 0.001), and CVD mortality (HR = 0.798, 95% CI: 0.691–0.921; *p* = 0.002). However, the corresponding positive coefficients for the PNI × log(time) term indicated that these inverse associations attenuated over time for all‐cause mortality (HR = 1.057, 95% CI: 1.036–1.079; *p* < 0.001), cancer mortality (HR = 1.068, 95% CI: 1.034–1.103; *p* < 0.001), non‐cancer mortality (HR = 1.052, 95% CI: 1.026–1.079; *p* < 0.001), and CVD mortality (HR = 1.0377, 95% CI: 1.002–1.079; *p* = 0.039). Similarly, PA showed a clear inverse association with mortality.

Similarly, an increase in PA was associated with a clear dose–response reduction in mortality risk. Compared with inactive participants, sufficient PA was associated with lower risks of all‐cause mortality (HR = 0.395, 95% CI: 0.277–0.564; *p* < 0.001), cancer mortality (HR = 0.434, 95% CI: 0.243–0.774; *p* = 0.005), non‐cancer mortality (HR = 0.377, 95% CI: 0.240–0.593; *p* < 0.001), and CVD mortality (HR = 0.374, 95% CI: 0.180–0.779; *p* = 0.009). Insufficient PA was also associated with lower risks of all‐cause mortality (HR = 0.637, 95% CI: 0.525–0.772; *p* < 0.001), cancer mortality (HR = 0.643, 95% CI: 0.462–0.895; *p* = 0.009), and non‐cancer mortality (HR = 0.637, 95% CI: 0.502–0.809; *p* < 0.001), but not CVD mortality (HR = 0.794, 95% CI: 0.547–1.151; *p* = 0.223). Overall, higher PA and higher PNI were associated with more favorable survival among cancer survivors, although the association between PNI and mortality weakened over time (Table 2).

**TABLE 2:** Association of PNI and PA with all‐cause, cancer, non‐cancer, and CVD mortality among US cancer survivors.

Mortality outcome	Model1		Model2		Model3	
	Hazard ratio (95% CI)	*p*	Hazard ratio (95% CI)	*p*	Hazard ratio (95% CI)	*p*
**All‐cause mortality**						
PNI						
Per 1‐unit increase	0.941 (0.925–0.958)	< 0.001	0.941 (0.925–0.958)	< 0.001	0.762 (0.704–0.823)	< 0.001
PNI × log(time)					1.057 (1.036–1.079)	< 0.001
PA						
Inactive	Reference		Reference		Reference	
Insufficiently active	0.568 (0.471–0.684)	< 0.001	0.610 (0.504–0.737)	< 0.001	0.637 (0.525–0.772)	< 0.001
Sufficiently active	0.348 (0.245–0.494)	< 0.001	0.364 (0.256–0.517)	< 0.001	0.395 (0.277–0.564)	< 0.001
**Cancer mortality**						
PNI						
Per 1‐unit increase	0.946 (0.918–0.974)	< 0.001	0.946 (0.918–0.975)	< 0.001	0.741 (0.655–0.839)	< 0.001
PNI × log(time)					1.068 (1.034–1.103)	< 0.001
PA						
Inactive	Reference		Reference		Reference	
Insufficiently active	0.583 (0.424–0.803)	< 0.001	0.621 (0.449–0.861)	0.004	0.643 (0.462–0.895)	0.009
Sufficiently active	0.385 (0.217–0.680)	0.001	0.404 (0.227–0.717)	0.002	0.434 (0.243–0.774)	0.005
**Non‐cancer mortality**						
PNI						
Per 1‐unit increase	0.939 (0.919–0.959)	< 0.001	0.939 (0.919–0.959)	< 0.001	0.771 (0.697–0.853)	< 0.001
PNI × log(time)					1.052 (1.026–1.079)	< 0.001
PA						
Inactive	Reference		Reference		Reference	
Insufficiently active	0.560 (0.446–0.705)	< 0.001	0.603 (0.477–0.762)	< 0.001	0.637 (0.502–0.809)	< 0.001
Sufficiently active	0.330 (0.211–0.514)	< 0.001	0.345 (0.221–0.540)	< 0.001	0.377 (0.240–0.593)	< 0.001
**CVD mortality**						
PNI						
Per 1‐unit increase	0.930 (0.897–0.963)	< 0.001	0.927 (0.894–0.960)	< 0.001	0.798 (0.691–0.921)	0.002
PNI × log(time)					1.040 (1.002–1.079)	0.039
PA						
Inactive	Reference		Reference		Reference	
Insufficiently active	0.679 (0.476–0.968)	0.032	0.694 (0.483–0.998)	0.049	0.794 (0.547–1.151)	0.223
Sufficiently active	0.347 (0.169–0.714)	0.004	0.346 (0.168–0.716)	0.004	0.374 (0.180–0.779)	0.009

*Note:* inactive, 0 MET‐min/week; insufficiently active, 1–599 MET‐min/week; sufficiently active, ≥ 600 MET‐min/week. Model 1 was an unadjusted conventional Cox proportional hazards model. Model 2 was a conventional Cox proportional hazards model adjusted for age, sex, race, marital status, education level, and PIR. Model 3 was an extended Cox model with time‐varying coefficients, additionally adjusted for smoking status, alcohol use, BMI, hypertension, hyperlipidemia, diabetes, obesity, abdominal obesity, and cancer subtype. Time‐varying terms were incorporated for PNI and age using interaction terms with log‐transformed follow‐up time. *p*‐value < 0.05 was considered significant.

Abbreviations: CVD mortality: cardiovascular disease mortality; PNI: Prognostic Nutritional Index; PA: physical activity; CI: confidence interval.

### 3.3 Dose–Response Pattern of PNI

**FIGURE 2:**
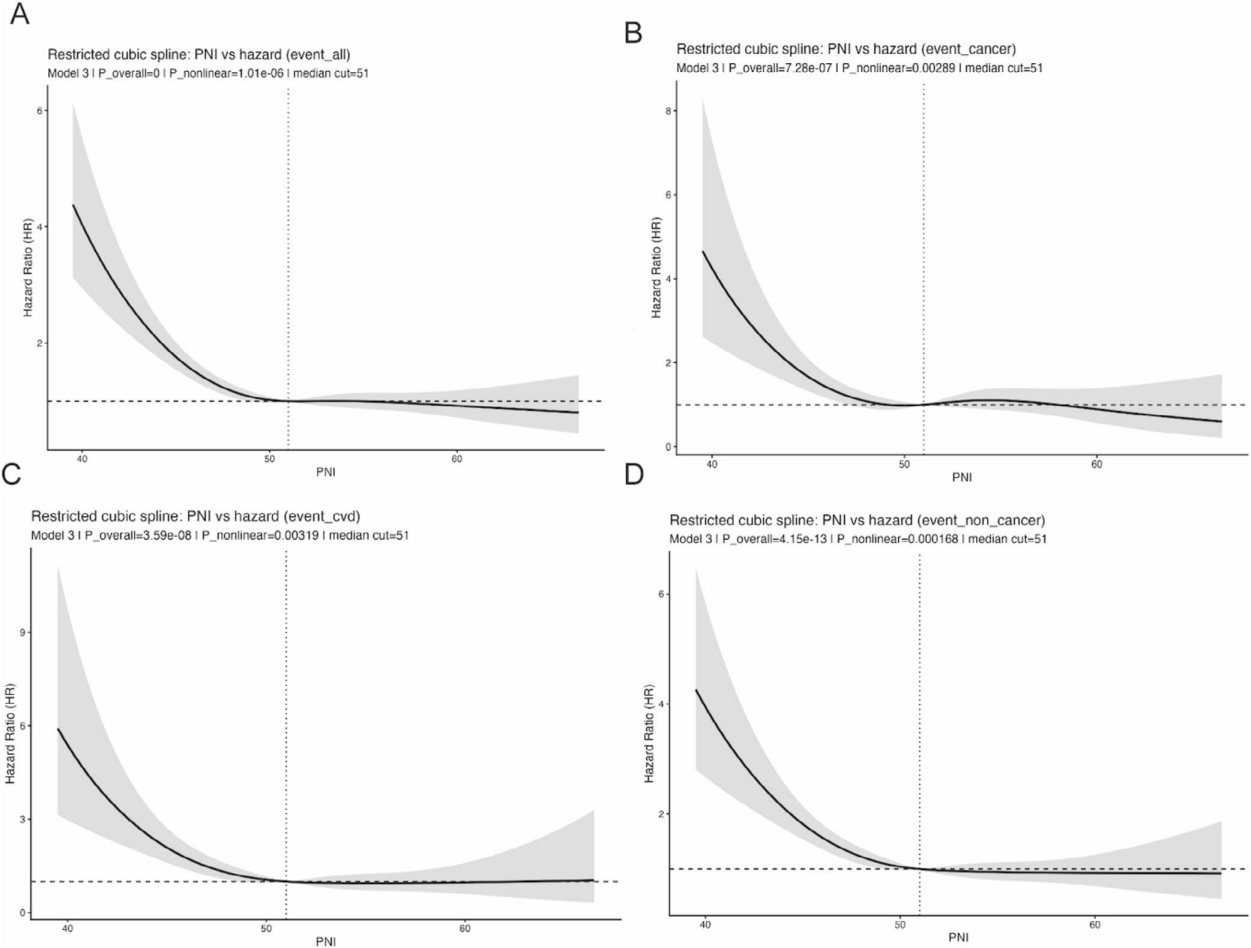
Restricted cubic spline (RCS) analyses of Prognostic Nutritional Index (PNI) and mortality outcomes among cancer survivors. (A) all‐cause mortality (B) cancer‐specific mortality (C) cardiovascular mortality (D) non‐cancer mortality.

Restricted cubic spline analyses based on the fully adjusted model demonstrated significant nonlinear relationships between continuous PNI and mortality outcomes, including all‐cause, cancer‐specific, CVD, and non‐cancer mortality (all *p* for overall association < 0.001; *p* for nonlinearity < 0.01) (Figure 2). Generally, lower PNI values were associated with a higher mortality risk, which declined as PNI increased.

### 3.4 Combined Analysis of PA and PNI With Mortality

In combined analyses, participants with high PNI (> 51) and sufficient PA showed the lowest risk of all‐cause mortality (HR = 0.30, 95% CI: 0.17–0.52; *p* < 0.01). Notably, among participants with low PNI, sufficient PA was also associated with lower all‐cause mortality (HR = 0.36, 95% CI: 0.23–0.56; *p* < 0.01). For non‐cancer mortality, high PNI combined with sufficient PA was linked to an approximately 78% lower risk (HR = 0.22, 95% CI: 0.10–0.49; *p* < 0.01). Similarly, among those with low PNI, sufficient PA corresponded to an estimated 43% reduction in non‐cancer mortality (HR = 0.575, 95% CI: 0.436–0.757; *p* < 0.01).

**FIGURE 3:**
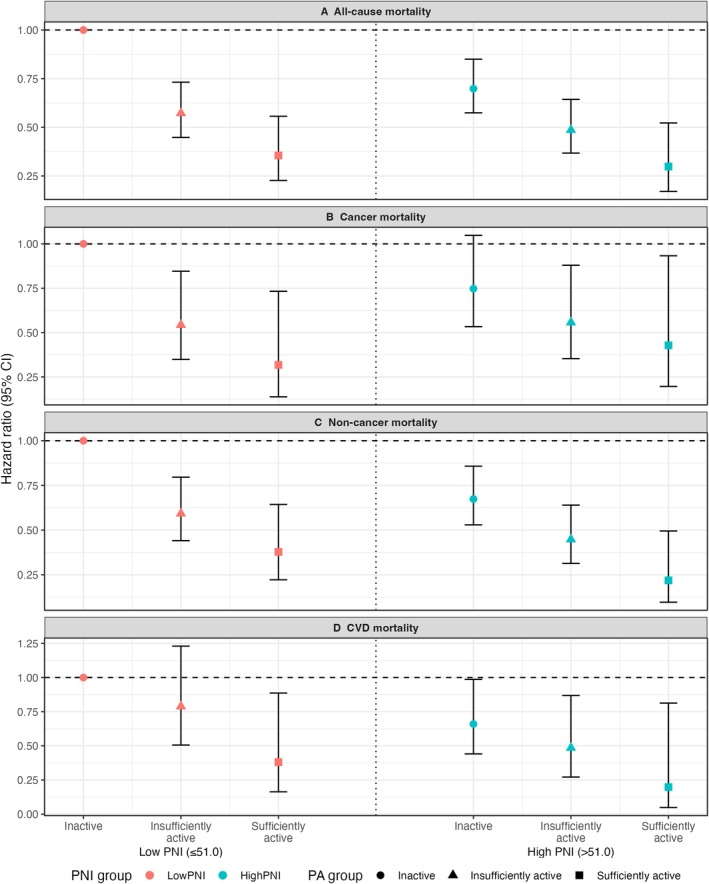
Joint association of PNI category and PA with mortality outcomes among cancer survivors. PNI; Prognostic Nutritional Index; PA, physical activity; CI, confidence interval; inactive, 0 MET‐min/week; insufficiently active, 1–599 MET‐min/week; sufficiently active, ≥ 600 MET‐min/week.

**FIGURE 4:**
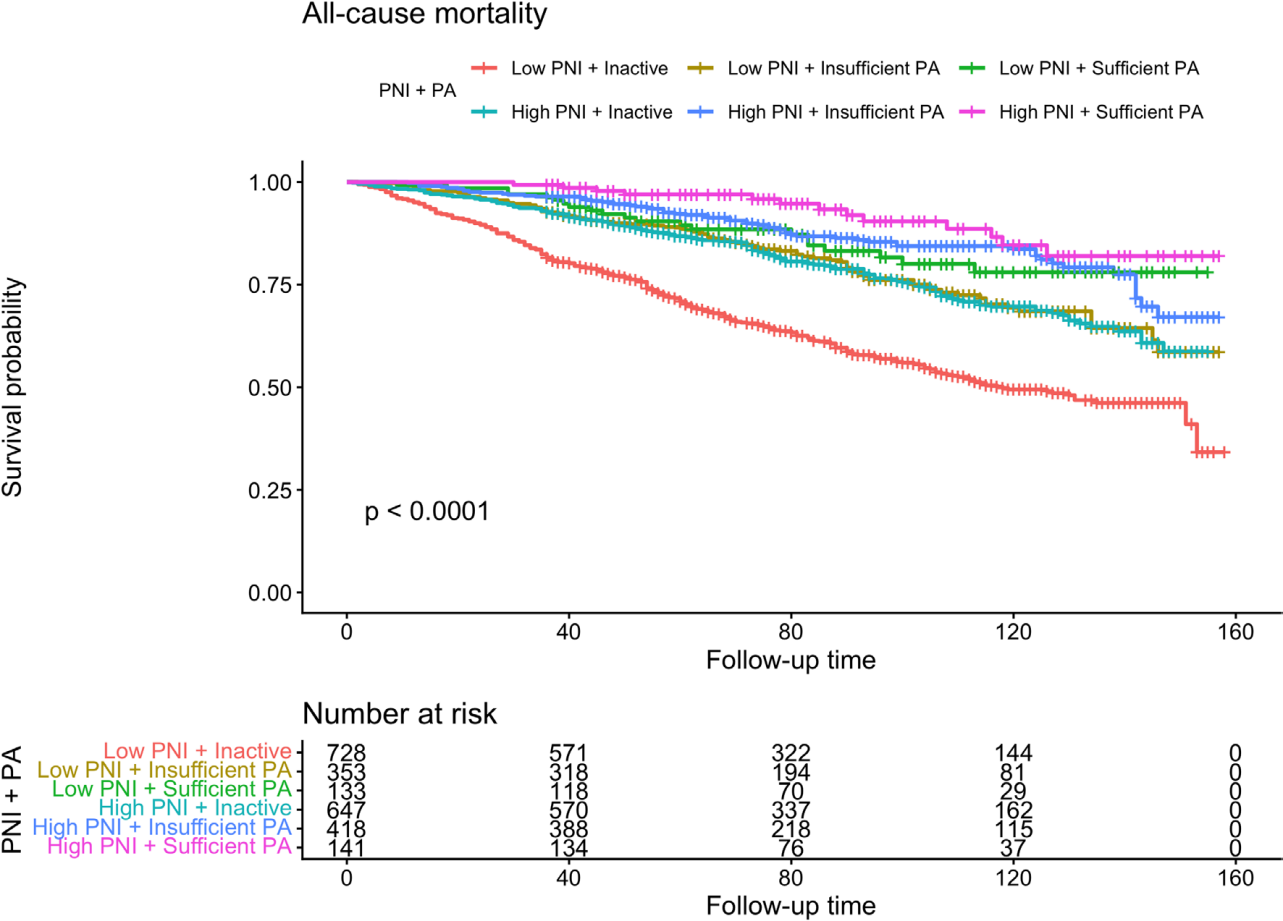
Kaplan–Meier overall survival curves by combined PNI and PA categories. PNI, Prognostic Nutritional index; PA, physical activity; CI, confidence interval.; PNI (low: ≤ 51; high: > 51); inactive, 0 MET‐min/week; insufficiently active, 1–599 MET‐min/week; sufficiently active, ≥ 600 MET‐min/week.

Sufficient PA was associated with a lower risk of cancer‐specific mortality across both PNI strata. Compared with inactive participants, cancer mortality was lower among sufficiently active individuals with high PNI (HR = 0.559, 95% CI: 0.344–0.908; *p* = 0.019) as well as among those with low PNI (HR = 0.470, 95% CI: 0.330–0.671; *p* < 0.01). For CVD mortality, the high PNI and sufficient PA group showed a lower point estimate, but the association was not statistically significant (Figure 3; Table S1). Supporting these findings, Kaplan–Meier curves showed clear separation in overall survival across the six combined PNI–PA groups (log‐rank *p* < 0.0001) (Figure 4). The low PNI and inactive group had the poorest survival, whereas the high PNI and sufficient PA group had the most favorable survival throughout follow‐up.

Collectively, these findings suggest complementary associations of PNI and PA with survival among cancer survivors.

### 3.5 Interaction Analyses of PA and PNI With Mortality

On the multiplicative scale, we found no evidence that PA modified the association between continuous PNI and mortality outcomes in the fully adjusted model, as likelihood ratio tests for the PNI × PA group interaction terms were not significant (Table S2). On the additive scale, a significant interaction was observed for all‐cause mortality (RERI = 0.52, 95% CI: 0.09–0.94; AP = 0.23, 95% CI: 0.04–0.40; SI = 1.69, 95% CI: 1.08–4.63) (Table S3), indicating an increased risk of mortality associated with the combined presence of low PNI and physical inactivity, which is greater than the sum of the risks associated with each factor individually. However, additive interaction estimates for cancer, non‐cancer, and CVD mortality were not statistically significant.

### 3.6 Subgroup Analysis

In the subgroup analysis of all‐cause mortality, significant associations were demonstrated between the high PNI group and sex, economic status, hypertension, obesity, diabetes, cancer category and cancer subtype. These associations were more pronounced in the sufficiently PA and high PNI subgroup, with the following HRs and 95% CIs: male participants (HR: 0.38; 95% CI: 0.21–0.67, *p* < 0.001), non‐impoverished participants (HR: 0.32; 95% CI: 0.18–0.57, *p* < 0.001), hypertensive participants (HR: 0.30; 95% CI: 0.16–0.57, *p* < 0.001), obese participants (HR: 0.33; 95% CI: 0.13–0.81, *p* = 0.016), non‐diabetic participants (HR: 0.24; 95% CI: 0.12–0.49, *p* < 0.001), obesity‐related cancer participants (HR: 0.13; 95% CI: 0.03–0.54 *p* = 0.005) and the prostate cancer participants (HR: 0.37; 95% CI: 0.14–0.97 *p* = 0.042). Results for subgroup analyses are presented in Table S4.

### 3.7 Sensitivity Analysis

Sensitivity analyses using alternative PNI categorizations produced consistent results PNI tertiles (with cutpoints at 49.5 and 53.5) showed a stepwise decrease in the risk of all‐cause, non‐cancer, CVD, and cancer mortality from T1 to T3 (all *p* ≤ 0.017). Similarly, median‐based dichotomization (with a cutpoint of 51) showed that high PNI was associated with lower all‐cause, non‐cancer, and CVD mortality (all *p* ≤ 0.008). These consistent findings across tertile‐ and median‐based classifications support the robustness of the observed associations and the appropriateness of the PNI categorization in this study (Tables S5 and S6).

### 3.8 Assessment of Proportional Hazards

Schoenfeld residual–based tests (Tables S7 and S8 and Figure S1) provided evidence of nonproportional hazards for PNI across outcomes (all‐cause: *p* = 1.32 × 10^−5^; cancer: *p* = 0.0037; non‐cancer: *p* = 0.0006) and for age in the all‐cause model (*p* = 0.0011). In contrast, PA did not show a clear violation of the proportional hazards assumption (*p* ≥ 0.05), although results were borderline in the all‐cause and non‐cancer models. Sensitivity analyses using extended Cox models with time‐varying coefficients for PNI and age produced directionally consistent estimates, supporting the robustness of the primary conclusions (Table S9).

## 4 Discussion

This study used data from the NHANES database to systematically evaluate the independent and joint associations of PNI and PA with the risk of mortality among cancer survivors. Our findings highlight the prognostic relevance of PNI. However, the extended Cox models further showed that this inverse association attenuated over time, indicating that the protective effect of baseline PNI was not constant during follow‐up. Higher PA was also associated with lower mortality risk, especially for all‐cause, cancer, and non‐cancer mortality. In the joint analysis, participants with high PNI and sufficient PA had the most favorable survival profile. Although no multiplicative interaction was detected, the additive interaction results suggested excess all‐cause mortality risk related to the coexistence of low PNI and physical inactivity.

These findings are consistent with previous research on PA and PNI in cancer rehabilitation. Prospective cohort studies suggest that higher levels of PA are associated with a lower risk of recurrence and mortality among cancer survivors [24]. Meanwhile, PNI—a composite indicator of nutritional and immune status—has been shown to predict postoperative complications and overall survival across multiple cancer types [25]. However, most previous studies have examined single‐component interventions (e.g., exercise prescriptions or nutritional supplementation), with little consideration of the potential interaction effects between PA and PNI [26]. Using NHANES data, this study provides evidence of a joint association of PNI and PA with mortality risk, supporting the development of multidimensional intervention strategies for cancer survivorship.

PNI comprises serum albumin and lymphocyte count, which effectively reflect the body's nutritional reserves and immune function status [27]. A higher PNI generally indicates adequate protein levels and preserved lymphocyte counts, suggesting improved immune surveillance and tissue repair capacity. Among cancer survivors, maintaining adequate nutritional and immune status can reduce the risk of recurrence and complications, thereby improving long‐term survival [28].

Mechanistically, PA may exert antitumor effects and improve overall health through multiple physiological pathways. Regular exercise improves cardiopulmonary function, enhances insulin sensitivity, and modulates chronic inflammation [29]. In addition, PA supports muscle protein synthesis, improves body composition, and enhances metabolic efficiency [30, 31]. Moreover, moderate exercise has been associated with reduced anxiety and depressive symptoms, as well as improved psychological resilience [13]. PA has also been associated with enhanced natural killer (NK) cell and T‐cell activity [32] and lower levels of pro‐inflammatory markers (e.g., C‐reactive protein and IL‐6) [33], which may reduce the risk of cancer recurrence [34]. Even when PNI levels are low, and there are potential deficits in nutritional and immune function, increasing PA can mitigate the adverse effects through these mechanisms. Moreover, when both PNI and PA levels are high, their combined effect is consistent with the expected sum of their independent effects, resulting in lower risks of all‐cause and cancer‐specific mortality.

Subgroup analyses suggested that sufficient PA was associated with lower mortality across groups, potentially varying by metabolic status, diet, and health behaviors. Socioeconomic disadvantage may be linked to malnutrition and lower PNI. Poor dietary quality over a prolonged period may reduce the health benefits of PA [35]. Additionally, limited access to healthcare may perpetuate sustained inflammation and weaken the associations between PA/PNI and mortality [36]. Beyond socioeconomic differences, sex‐specific physiological factors may also modify the associations of PNI and PA with mortality. Males typically exhibit a higher basal metabolic rate and greater skeletal muscle mass, which could make them more responsive to PA. Among individuals with high PNI, PA further enhances muscle synthesis, improves insulin sensitivity, and reduces inflammation levels [37].

In individuals with chronic diseases, the joint associations of PA and PNI with mortality appear more pronounced. PA has been associated with a lower risk of CVD in patients with hypertension, potentially via reduced vascular resistance, increased nitric oxide (NO) bioavailability, and improved arterial elasticity [38]. Similarly, higher PNI may indicate better protein and antioxidant reserves, which could help to reduce oxidative stress and inflammation associated with hypertension [39]. In diabetes‐ and obesity‐stratified analyses, higher PNI was consistently associated with lower mortality, suggesting that better nutritional–immune status may partially offset baseline risk. In diabetes, this pattern may be related to metabolic dysfunction, systemic inflammation, and cardiovascular comorbidity [40]. Phillips et al. reported that higher PNI in diabetes is associated with better protein nutritional status, which may support PA‐related benefits (e.g., muscle mass preservation, lower inflammation, and improved metabolic control) [41]. However, if PA is insufficient, the benefit associated with higher PNI may be attenuated, given links between low PA, muscle loss, glycemic instability, and chronic inflammation [42]. Obesity is often accompanied by insulin resistance, chronic inflammation, and elevated cardiovascular risk. PA may improve metabolic health through increased energy expenditure, enhanced fat oxidation, and reduced visceral adiposity [43]. Consistent with this, among survivors with obesity‐related cancers, each 1‐point increase in PA was associated with a 12% lower risk of all‐cause mortality [44]. Previous studies also reported that higher post‐diagnosis PA is associated with better overall and prostate cancer‐specific survival, and that PNI provides prognostic stratification in prostate cancer [31, 45]. These observations are consistent with our results.

Notably, we observed a significant association for PNI with CVD mortality when examined individually, whereas the joint model did not show a significant association with CVD mortality. This discrepancy may be due to competing risks, given that as cancer progression and treatment‐related complications account for a large proportion of deaths in cancer survivors, resulting in fewer CVD deaths in our sample. In addition, different PA modalities may have different associations with cardiovascular risk, and a longer follow‐up period may be necessary to clarify these relationships [46, 47].

## 5 Strengths and Limitations

Several strengths should be noted. Firstly, this analysis used a large, nationally representative dataset, which enhances the generalizability of the findings. Secondly, by jointly evaluating PA and PNI, the study provides an integrated perspective linking exercise, nutrition, and immune status in cancer survivorship, consistent with cross‐disciplinary concepts such as “nutrition–immunity–metabolism” and “exercise–inflammation regulation.” Thirdly, the results could inform risk stratification and support the development of multidimensional rehabilitation plans in clinical and public health settings, incorporating comprehensive approaches that combine nutritional support with exercise‐based interventions.

Nevertheless, there are several limitations that warrant consideration. The observational design precludes causal inference; therefore, prospective cohorts and randomized trials are needed to clarify mechanisms and to determine whether improving PNI and PA translates into better survival across cancer types. PA was self‐reported and may be subject to measurement error; more accurate estimates of PA volume and intensity could be provided by wearable devices or accelerometry. Although multiple imputation was used to address missing covariates, exclusions required to define the analytical cohort (e.g., missing exposure information) may introduce selection bias and limit generalizability. In addition, cancer history in NHANES is self‐reported rather than registry‐validated, which could lead to misclassification and potentially weaken the observed associations. Moreover, detailed cancer stage and treatment information was not consistently available, and the limited sample sizes for less common subtypes restricted cancer‐type–specific inference. PNI only captures part of nutritional and immune status, so future studies should incorporate inflammatory biomarkers, muscle mass, and other nutritional indicators. Finally, as associations may differ by exercise type and intensity (e.g., aerobic vs. resistance training), further stratified analyses are warranted. Moreover, some subgroup strata contained zero events in certain joint exposure categories, which can yield unstable Cox regression estimates.

## 6 Conclusions

In summary, this large population‐based study suggests that higher PNI and higher PA are jointly associated with lower mortality risk among cancer survivors. These findings not only provide new insights for clinical and public health interventions but also lay the foundation for further exploration of the integrated mechanisms linking “nutrition–immunity–exercise–cancer.” High‐quality prospective studies in the future are urgently needed to validate and refine these conclusions, thereby continuously improving the evidence base for cancer rehabilitation management and health interventions.

## Author Contributions

**Linli Chen:** conceptualization, methodology, validation, writing – original draft, writing – review and editing. **Yinhao Chen:** conceptualization, formal analysis, visualization. **Yuhan Li:** investigation. **Yutao Li:** software, data curation. **Xiang Ruan:** resources. **Ingo G. H. Schmidt‐Wolf:** supervision, project administration, funding acquisition. **Paula Tups:** supervision, project administration. All authors have read and agreed to the published version of the manuscript.

## Funding

The authors have nothing to report.

## Ethics Statement

The NHANES study protocols were reviewed and approved by the National Center for Health Statistics Research Ethics Review Board. For the NHANES cycles included in this study, the 2007–2010 cycles were approved under Continuation of Protocol #2005‐06, and the 2011–2016 cycles were approved under Protocol/Continuation of Protocol #2011‐17. Written informed consent was obtained from all participants before data collection. As this study used publicly available and de‐identified NHANES data, additional institutional review board approval was not required.

This investigation is governed by an ethical framework that aligns with standardized public health research protocols. Data acquisition for NHANES was administered by CDC, with study protocols receiving formal approval from the National Center for Health Statistics (NCHS) Institutional Review Board. Written informed consent was obtained from all enrolled subjects prior to data collection. As this analysis exclusively utilized de‐identified publicly accessible datasets, there was no direct human experimentation or exposure of personally identifiable information, thereby exempting the study from supplementary ethical clearance requirements. The research was conducted in strict accordance with the ethical principles outlined in the Declaration of Helsinki, ensuring alignment with international biomedical research norms. Methodological rigor included full compliance with NHANES data governance policies, federal regulatory statutes, and cybersecurity protocols to safeguard participant confidentiality and dataset integrity.

## Consent

Informed consent was obtained from all subjects involved at the time of data collection for the NHANES 2007–2016. Patient consent was waived for the present study due to the use of publicly available data.

## Conflicts of Interest

The authors declare no conflicts of interest.

## Supporting information


**Figure S1:** cam471767‐sup‐0001‐FigureS1.png.

**Table S1:** Joint association of PNI and PA with all‐cause, cancer, non‐cancer, and CVD mortality among US cancer survivors (NHANES 2007–2016).
**Table S2:** Multiplicative interaction between continuous PNI and PA categories (inactive/insufficiently active/sufficiently active) on mortality outcomes (Model 3).
**Table S3:** Additive interaction between PNI category (low vs high; median split) and PA (inactive vs active) on mortality outcomes (Model 3).
**Table S4:** The subgroup analysis of PA combined with PNI with all‐cause mortality, cancer‐related mortality, and non‐cancer‐related mortality among US cancer survivors.
**Table S5:** Sensitivity analysis using PNI tertiles in Model 3.
**Table S6:** Sensitivity analysis using PNI dichotomized at the median (cutpoint = 51) in Model 3.
**Table S7:** Proportional hazards (PH) assumption assessment for Model 3 using Schoenfeld residual tests (key terms).
**Table S8:** Proportional hazards (PH) assumption assessment for Model 3 (all covariates).
**Table S9:** Sensitivity analysis using an extended Cox model with time‐varying coefficients for PNI and age (Model 3).

## Data Availability

The data that support the findings of this study are available in National Health and Nutrition Examination Survey (NHANES), at https://www.cdc.gov/nchs/nhanes/index.htm. These data were derived from the following resources available in the public domain: CDC website, https://www.cdc.gov/nchs/nhanes.
