# Supplementary figures and images for: Joint Association of Physical Activity and Prognostic Nutritional Index on Survival in US Cancer Survivors: A Study Based on the NHANES Database
Source: Cancer Med. 2026 May 10;15(5):e71767. doi: 10.1002/cam4.71767 (PMC13158273; doi:10.1002/cam4.71767)

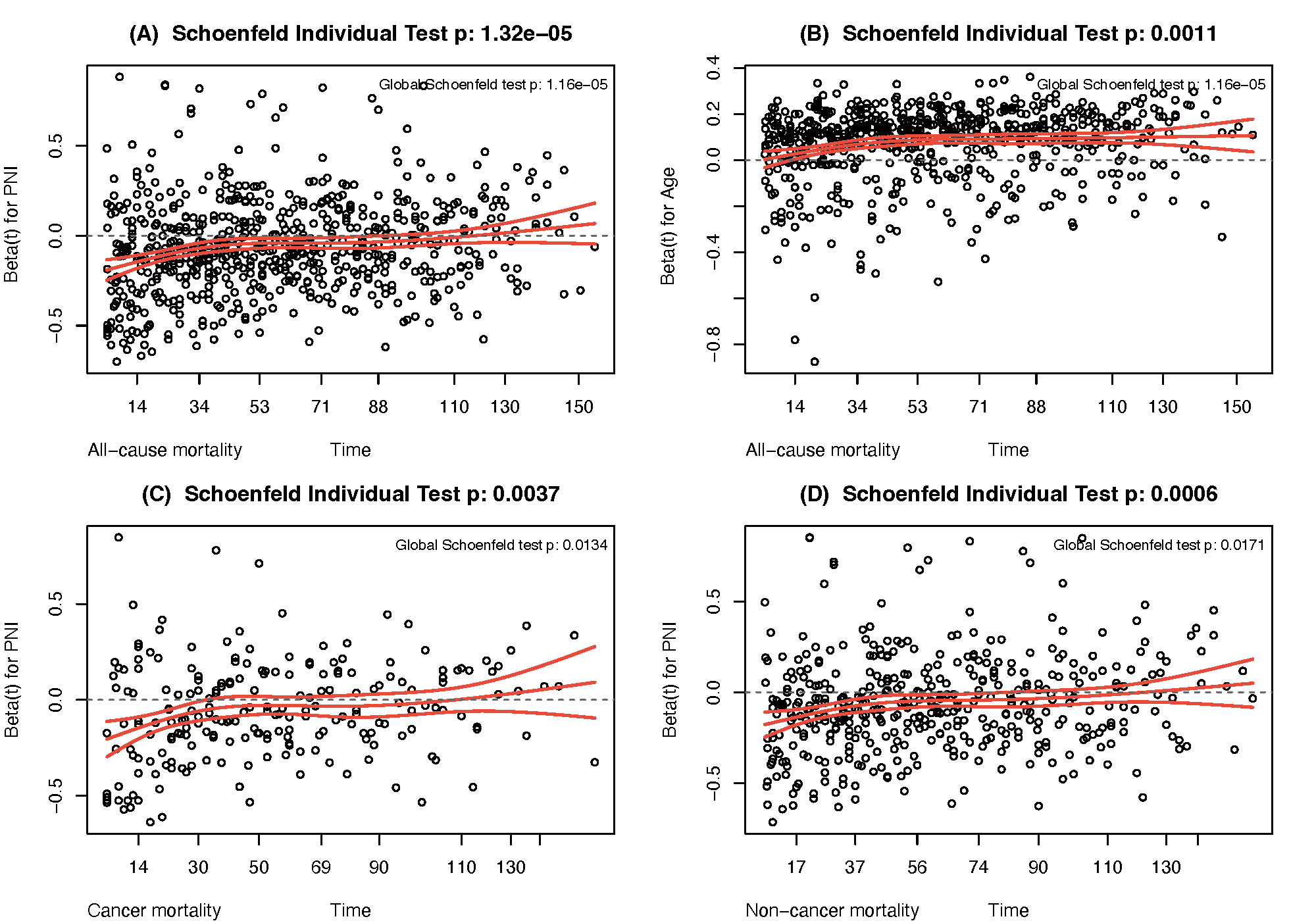

Supplement: Supplementary file 1 — Figure S1: cam471767‐sup‐0001‐FigureS1.png. [file CAM4-15-e71767-s002.png]
