# Supplementary material for: Joint Association of Physical Activity and Prognostic Nutritional Index on Survival in US Cancer Survivors: A Study Based on the NHANES Database
Source: Cancer Med. 2026 May 10;15(5):e71767. doi: 10.1002/cam4.71767 (PMC13158273; doi:10.1002/cam4.71767)
Supplement: Supplementary file 2 — Table S1: Joint association of PNI and PA with all‐cause, cancer, non‐cancer, and CVD mortality among US cancer survivors (NHANES 2007–2016). Table S2: Multiplicative interaction between continuous PNI and PA categories (inactive/insufficiently active/sufficiently active) on mortality outcomes (Model 3). Table S3: Additive interaction between PNI category (low vs high; median split) and PA (inactive vs active) on mortality outcomes (Model 3). Table S4: The subgroup analysis of PA combined with PNI with all‐cause mortality, cancer‐related mortality, and non‐cancer‐related mortality among US cancer survivors. Table S5: Sensitivity analysis using PNI tertiles in Model 3. Table S6: Sensitivity analysis using PNI dichotomized at the median (cutpoint = 51) in Model 3. Table S7: Proportional hazards (PH) assumption assessment for Model 3 using Schoenfeld residual tests (key terms). Table S8: Proportional hazards (PH) assumption assessment for Model 3 (all covariates). Table S9: Sensitivity analysis using an extended Cox model with time‐varying coefficients for PNI and age (Model 3). [file CAM4-15-e71767-s001.docx]

**Supplementary files**

**Joint Association of Physical Activity and Prognostic Nutritional Index on Survival in U.S. Cancer Survivors: A Study Based on the NHANES Database**

Linli Chen^1#^, Yinhao Chen^1#^_,_ Yutao Li^1^, Yuhan Li^1^, Xiang Ruan^1^, Paula Tups^1^, Ingo G.H. Schmidt-Wolf^1*^

1. Department of Integrated Oncology, Center for Integrated Oncology (CIO), University Hospital Bonn, Bonn, Germany

#These authors contributed equally to this work.

*Correspondence:

Prof. Dr. med. Ingo G.H. Schmidt Wolf

Department of Integrated Oncology

University Hospital Bonn

Venusberg Campus 1, D 53127 Bonn, Germany

1. mail: ingo. [schmidt-wolf@ukbonn.de](mailto:schmidt-wolf@ukbonn.de)

**Table S1. Joint association of PNI and PA with all-cause, cancer, non-cancer, and CVD mortality among U.S. cancer survivors (NHANES 2007-2016)**

| Mortality outcome | Model1 | | Model2 | | Model3 | *P* |
| --- | --- | --- | --- | --- | --- | --- |
|  | Hazard ratio (95% CI) | *P* | Hazard ratio (95% CI) | *P* | Hazard ratio (95% CI) |  |
| **All-cause mortality** |  |  |  |  |  |  |
| Inactive and LowPNI | 1.00 (ref) |  | 1.00 (ref) |  | 1.00 (ref) |  |
| Inactive and HighPNI | 0.50 (0.41-0.60) | <0.001 | 0.73 (0.60-0.88) | 0.001 | 0.70 (0.57-0.85) | <0.001 |
| Insufficiently active and HighPNI | 0.29 (0.22-0.38) | <0.001 | 0.47 (0.36-0.62) | <0.001 | 0.49 (0.37-0.64) | <0.001 |
| Insufficiently active and LowPNI | 0.48 (0.38-0.61) | <0.001 | 0.57 (0.45-0.73) | <0.001 | 0.57 (0.45-0.73) | <0.001 |
| Sufficiently active and HighPNI | 0.18 (0.10-0.31) | <0.001 | 0.29 (0.17-0.50) | <0.001 | 0.30 (0.17-0.52) | <0.001 |
| Sufficiently active and LowPNI | 0.32 (0.21-0.50) | <0.001 | 0.33 (0.21-0.51) | <0.001 | 0.36 (0.23-0.56) | <0.001 |
| **Cancer mortality** |  |  |  |  |  |  |
| Inactive and LowPNI | 1.00 (ref) |  | 1.00 (ref) |  | 1.00 (ref) |  |
| Inactive and HighPNI | 0.59 (0.43-0.82) | 0.002 | 0.78 (0.55-1.09) | 0.139 | 0.75 (0.53-1.05) | 0.092 |
| Insufficiently active and HighPNI | 0.39 (0.25-0.61) | <0.001 | 0.55 (0.35-0.86) | 0.009 | 0.56 (0.35-0.88) | 0.012 |
| Insufficiently active and LowPNI | 0.49 (0.32-0.75) | 0.001 | 0.55 (0.35-0.85) | 0.007 | 0.54 (0.35-0.85) | 0.007 |
| Sufficiently active and HighPNI | 0.32 (0.15-0.69) | 0.004 | 0.44 (0.20-0.95) | 0.036 | 0.43 (0.20-0.93) | 0.033 |
| Sufficiently active and LowPNI | 0.31 (0.13-0.70) | 0.005 | 0.29 (0.13-0.67) | 0.004 | 0.32 (0.14-0.73) | 0.007 |
| **Noncancer mortality** | 0.939 (0.919-0.959) | <0.001 | 0.939 (0.919-0.959) | <0.001 | 0.938 (0.918-0.958) | <0.001 |
| Inactive and LowPNI | 1.00 (ref) |  | 1.00 (ref) |  | 1.00 (ref) |  |
| Inactive and HighPNI | 0.45 (0.36-0.58) | <0.001 | 0.70 (0.55-0.89) | 0.004 | 0.67 (0.53-0.86) | 0.001 |
| Insufficiently active and HighPNI | 0.25 (0.18-0.35) | <0.001 | 0.43 (0.30-0.61) | <0.001 | 0.45 (0.31-0.64) | <0.001 |
| Insufficiently active and LowPNI | 0.48 (0.36-0.63) | <0.001 | 0.58 (0.43-0.78) | <0.001 | 0.59 (0.44-0.80) | <0.001 |
| Sufficiently active and HighPNI | 0.12 (0.05-0.26) | <0.001 | 0.21 (0.09-0.47) | <0.001 | 0.22 (0.10-0.49) | <0.001 |
| Sufficiently active and LowPNI | 0.33 (0.20-0.56) | <0.001 | 0.35 (0.21-0.59) | <0.001 | 0.38 (0.22-0.64) | <0.001 |
| **CVD mortality** |  |  |  |  |  |  |
| Inactive and LowPNI | 1.00 (ref) |  | 1.00 (ref) |  | 1.00 (ref) |  |
| Inactive and HighPNI | 0.43 (0.29-0.63) | <0.001 | 0.69 (0.46-1.03) | 0.069 | 0.66 (0.44-0.99) | 0.043 |
| Insufficiently active and HighPNI | 0.25 (0.14-0.44) | <0.001 | 0.44 (0.24-0.78) | 0.005 | 0.49 (0.27-0.87) | 0.015 |
| Insufficiently active and LowPNI | 0.60 (0.39-0.92) | 0.02 | 0.70 (0.45-1.09) | 0.118 | 0.79 (0.51-1.23) | 0.296 |
| Sufficiently active and HighPNI | 0.10 (0.03-0.43) | 0.002 | 0.18 (0.04-0.72) | 0.016 | 0.20 (0.05-0.81) | 0.025 |
| Sufficiently active and LowPNI | 0.35 (0.15-0.80) | 0.013 | 0.36 (0.16-0.84) | 0.017 | 0.38 (0.16-0.89) | 0.025 |

CVD mortality, cardiovascular disease mortality; PNI: prognostic nutritional index; CI, confidence interval

Model 1 was unadjusted; Model 2 adjusted for age, sex, race, marital status, education level, and PIR; Model 3 further adjusted for smoking status, alcohol consumption, BMI, hypertension, hyperlipidemia, diabetes, obesity, abdominal obesity, and cancer subtype

Low: PNI≤51.0; High: PNI>51.0

Inactive: 0 MET-min/week. Insufficiently active: 1-599 MT-min/week. Sufficiently active: ≥600 MET-min/week

*P*-value<0.05 was considered significant.

**Table S2. Multiplicative interaction between continuous PNI and PA categories (inactive/insufficiently active/sufficiently active) on mortality outcomes (Model 3)**

| Outcome | Model | LRT chi-square | df | *P* for interaction |
| --- | --- | --- | --- | --- |
| All-cause | Model3 | 2.214666226 | 2 | 0.330439031 |
| Cancer | Model3 | 0.573283625 | 2 | 0.750780601 |
| Non-cancer | Model3 | 1.5221251 | 2 | 0.467169772 |
| CVD | Model3 | 1.717885228 | 2 | 0.423609765 |

CVD, cardiovascular disease；LRT: Likelihood Ratio Test；df: degrees of freedom

P values < 0.05 indicate evidence against proportional hazards.

**Table S3 Additive interaction between PNI category (low vs high; median split) and PA (inactive vs active) on mortality outcomes (Model 3).**

| Outcome | Model | RERI (95% CI) | AP (95% CI) | SI (95% CI) | Bootstrap success | Bootstrap B |
| --- | --- | --- | --- | --- | --- | --- |
| All-cause | Model3 | 0.52 (0.09-0.94) | 0.23 (0.04-0.40) | 1.69 (1.08-4.63) | 1,000 | 1,000 |
| Cancer | Model3 | 0.57 (-0.16-1.22) | 0.30 (-0.08-0.60) | 2.64 (-16.70-18.89) | 1,000 | 1,000 |
| Non-cancer | Model3 | 0.47 (-0.18-1.04) | 0.19 (-0.06-0.40) | 1.44 (0.90-3.52) | 1,000 | 1,000 |
| CVD | Model3 | 0.21 (-0.98-1.22) | 0.09 (-0.38-0.48) | 1.17 (-1.06-5.10) | 1,000 | 1,000 |

RERI, relative excess risk due to interaction; AP, attributable proportion due to interaction; SI, synergy index; CI, confidence interval; CVD, cardiovascular disease

PNI was dichotomized at the median 51 (low vs high). PA was dichotomized as inactive vs active (active = insufficiently active or sufficiently active). Additive interaction was quantified using RERI, AP, and SI based on Model 3 estimates.

**Table S4. The subgroup analysis of PA combined with PNI with all-cause mortality, cancer-related mortality and non-cancer-related mortality among US cancer survivors.**

|  |  | All-cause mortality | | | | cancer-mortality | | | | no-cancer-mortality | | | |
| --- | --- | --- | --- | --- | --- | --- | --- | --- | --- | --- | --- | --- | --- |
| subgroup | groups | N/Events | HR (95%Cl) | p | *P* for interaction | N/Events | HR (95%Cl) | p | *P* for interaction | N/Events | HR (95%Cl) | p | *P* for interaction |
| sex |  |  |  |  | 0.087 |  |  |  | 0.609 |  |  |  | 0.229 |
| Male | Inactive and LowPNI | 350/187 | Reference |  |  | 350/61 | Reference |  |  | 350/126 | Reference |  |  |
|  | Sufficiently active and HighPNI | 73/13 | 0.38 (0.21–0.67) | <0.001 |  | 73/7 | 0.57 (0.26–1.28) | 0.175 |  | 73/6 | 0.27 (0.12–0.62) | 0.002 |  |
|  | Inactive and HighPNI | 275/82 | 0.68 (0.52–0.88) | 0.004 |  | 275/32 | 0.72 (0.46–1.11) | 0.139 |  | 275/50 | 0.66 (0.47–0.92) | 0.015 |  |
|  | Insufficiently active and HighPNI | 198/39 | 0.45 (0.32–0.65) | <0.001 |  | 198/18 | 0.56 (0.32–0.98) | 0.043 |  | 198/21 | 0.40 (0.25–0.65) | <0.001 |  |
|  | Sufficiently active and LowPNI | 89/19 | 0.41 (0.25–0.66) | <0.001 |  | 89/5 | 0.33 (0.13–0.82) | 0.017 |  | 89/14 | 0.46 (0.26–0.82) | 0.008 |  |
|  | Insufficiently active and LowPNI | 168/53 | 0.53 (0.38–0.72) | <0.001 |  | 168/18 | 0.56 (0.33–0.97) | 0.038 |  | 168/35 | 0.52 (0.35–0.77) | <0.001 |  |
| Female | Inactive and LowPNI | 378/126 | Reference |  |  | 378/34 | Reference |  |  | 378/92 | Reference |  |  |
|  | Sufficiently active and HighPNI | 68/0 | NE |  |  | 68/0 | NE |  |  | 68/0 | NE |  |  |
|  | Inactive and HighPNI | 372/74 | 0.65 (0.48–0.88) | 0.005 |  | 372/24 | 0.76 (0.44–1.31) | 0.325 |  | 372/50 | 0.61 (0.43–0.87) | 0.007 |  |
|  | Insufficiently active and HighPNI | 220/23 | 0.48 (0.30–0.75) | 0.002 |  | 220/7 | 0.45 (0.20–1.06) | 0.067 |  | 220/16 | 0.48 (0.28–0.83) | 0.009 |  |
|  | Sufficiently active and LowPNI | 44/2 | 0.14 (0.03–0.58) | 0.006 |  | 44/1 | 0.24 (0.03–1.76) | 0.16 |  | 44/1 | 0.11 (0.01–0.76) | 0.026 |  |
|  | Insufficiently active and LowPNI | 185/32 | 0.60 (0.40–0.89) | 0.011 |  | 185/8 | 0.56 (0.26–1.23) | 0.149 |  | 185/24 | 0.64 (0.40–1.01) | 0.054 |  |
| PIR |  |  |  |  | 0.375 |  |  |  | 0.22 |  |  |  | 0.423 |
| Not poor | Inactive and LowPNI | 616/268 | Reference |  |  | 616/81 | Reference |  |  | 616/187 | Reference |  |  |
|  | Sufficiently active and HighPNI | 126/12 | 0.32 (0.18–0.57) | <0.001 |  | 126/7 | 0.48 (0.22–1.04) | 0.064 |  | 126/13 | 0.21 (0.09–0.52) | <0.001 |  |
|  | Inactive and HighPNI | 521/127 | 0.68 (0.55–0.84) | <0.001 |  | 521/40 | 0.64 (0.44–0.95) | 0.026 |  | 521/87 | 0.70 (0.54–0.90) | 0.007 |  |
|  | Insufficiently active and HighPNI | 374/54 | 0.46 (0.34–0.62) | <0.001 |  | 374/21 | 0.49 (0.30–0.81) | 0.005 |  | 374/33 | 0.44 (0.30–0.64) | <0.001 |  |
|  | Sufficiently active and LowPNI | 123/19 | 0.33 (0.21–0.53) | <0.001 |  | 123/6 | 0.31 (0.13–0.72) | 0.007 |  | 123/13 | 0.35 (0.20–0.61) | <0.001 |  |
|  | Insufficiently active and LowPNI | 313/80 | 0.60 (0.46–0.77) | <0.001 |  | 313/24 | 0.55 (0.35–0.88) | 0.012 |  | 313/56 | 0.63 (0.46–0.85) | 0.003 |  |
| Poor | Inactive and LowPNI | 112/45 | Reference |  |  | 112/14 | Reference |  |  | 112/31 | Reference |  |  |
|  | Sufficiently active and HighPNI | 15/1 | 0.17 (0.02–1.39) | 0.098 |  | 15/0 | NE |  |  | 15/1 | 0.20 (0.02–1.85) | 0.158 |  |
|  | Inactive and HighPNI | 126/29 | 0.81 (0.49–1.34) | 0.407 |  | 126/16 | 1.59 (0.71–3.60) | 0.262 |  | 126/13 | 0.46 (0.23–0.93) | 0.03 |  |
|  | Insufficiently active and HighPNI | 44/8 | 0.82 (0.35–1.90) | 0.643 |  | 44/4 | 1.70 (0.50–5.84) | 0.399 |  | 44/4 | 0.53 (0.16–1.74) | 0.294 |  |
|  | Sufficiently active and LowPNI | 10/2 | 0.81 (0.18–3.54) | 0.775 |  | 10/0 | NE |  |  | 10/2 | 1.28 (0.27–6.03) | 0.753 |  |
|  | Insufficiently active and LowPNI | 40/5 | 0.35 (0.13–0.93) | 0.035 |  | 40/2 | 0.51 (0.11–2.43) | 0.402 |  | 40/3 | 0.30 (0.08–1.05) | 0.059 |  |
| hypertension |  |  |  |  | 0.648 |  |  |  | 0.24 |  |  |  | 0.771 |
| No | Inactive and LowPNI | 211/64 | Reference |  |  | 211/23 | Reference |  |  | 211/41 | Reference |  |  |
|  | Sufficiently active and HighPNI | 66/3 | 0.30 (0.09–0.99) | 0.048 |  | 66/1 | 0.20 (0.03–1.50) | 0.118 |  | 66/2 | 0.35 (0.08–1.54) | 0.166 |  |
|  | Inactive and HighPNI | 224/34 | 0.68 (0.44–1.06) | 0.087 |  | 224/15 | 0.76 (0.39–1.52) | 0.443 |  | 224/19 | 0.59 (0.32–1.07) | 0.084 |  |
|  | Insufficiently active and HighPNI | 178/17 | 0.61 (0.35–1.07) | 0.087 |  | 178/7 | 0.49 (0.20–1.20) | 0.121 |  | 178/10 | 0.58 (0.27–1.24) | 0.162 |  |
|  | Sufficiently active and LowPNI | 64/10 | 0.53 (0.27–1.07) | 0.077 |  | 64/3 | 0.34 (0.10–1.18) | 0.089 |  | 64/7 | 0.63 (0.27–1.50) | 0.299 |  |
|  | Insufficiently active and LowPNI | 117/17 | 0.53 (0.31–0.94) | 0.028 |  | 117/2 | 0.16 (0.04–0.67) | 0.013 |  | 117/15 | 0.83 (0.44–1.59) | 0.579 |  |
| Yes | Inactive and LowPNI | 517/249 | Reference |  |  | 517/72 | Reference |  |  | 517/177 | Reference |  |  |
|  | Sufficiently active and HighPNI | 75/10 | 0.30 (0.16–0.57) | <0.001 |  | 75/6 | 0.54 (0.23–1.26) | 0.154 |  | 75/4 | 0.18 (0.07–0.49) | <0.001 |  |
|  | Inactive and HighPNI | 423/122 | 0.69 (0.55–0.86) | 0.001 |  | 423/41 | 0.75 (0.51–1.12) | 0.158 |  | 423/81 | 0.66 (0.51–0.86) | 0.003 |  |
|  | Insufficiently active and HighPNI | 240/45 | 0.45 (0.33–0.63) | <0.001 |  | 240/18 | 0.59 (0.35–1.01) | 0.054 |  | 240/27 | 0.39 (0.26–0.59) | <0.001 |  |
|  | Sufficiently active and LowPNI | 69/11 | 0.27 (0.14–0.49) | <0.001 |  | 69/3 | 0.27 (0.08–0.85) | 0.025 |  | 69/8 | 0.27 (0.13–0.55) | <0.001 |  |
|  | Insufficiently active and LowPNI | 236/68 | 0.59 (0.44–0.77) | <0.001 |  | 236/24 | 0.71 (0.44–1.13) | 0.15 |  | 236/44 | 0.54 (0.38–0.75) | <0.001 |  |
| diabetes |  |  |  |  | 0.348 |  |  |  | 0.126 |  |  |  | 0.937 |
| No | Inactive and LowPNI | 497/191 | Reference |  |  | 497/58 | Reference |  |  | 497/133 | Reference |  |  |
|  | Sufficiently active and HighPNI | 119/8 | 0.24 (0.12–0.49) | <0.001 |  | 119/4 | 0.35 (0.13–0.98) | 0.046 |  | 119/4 | 0.18 (0.07–0.50) | <0.001 |  |
|  | Inactive and HighPNI | 460/96 | 0.78 (0.60–1.00) | 0.048 |  | 460/38 | 0.89 (0.59–1.36) | 0.601 |  | 460/58 | 0.72 (0.52–0.99) | 0.04 |  |
|  | Insufficiently active and HighPNI | 340/51 | 0.57 (0.41–0.78) | <0.001 |  | 340/23 | 0.79 (0.48–1.31) | 0.368 |  | 340/28 | 0.46 (0.30–0.70) | <0.001 |  |
|  | Sufficiently active and LowPNI | 99/12 | 0.31 (0.17–0.57) | <0.001 |  | 99/4 | 0.33 (0.12–0.92) | 0.034 |  | 99/8 | 0.31 (0.15–0.64) | 0.002 |  |
|  | Insufficiently active and LowPNI | 279/69 | 0.62 (0.47–0.83) | 0.001 |  | 279/23 | 0.67 (0.40–1.09) | 0.109 |  | 279/46 | 0.60 (0.43–0.86) | 0.005 |  |
| Yes | Inactive and LowPNI | 231/122 | Reference |  |  | 231/37 | Reference |  |  | 231/85 | Reference |  |  |
|  | Sufficiently active and HighPNI | 22/5 | 0.41 (0.17–1.03) | 0.059 |  | 22/3 | 0.64 (0.19–2.18) | 0.477 |  | 22/2 | 0.26 (0.06–1.06) | 0.061 |  |
|  | Inactive and HighPNI | 187/60 | 0.61 (0.44–0.83) | 0.002 |  | 187/18 | 0.54 (0.30–0.97) | 0.039 |  | 187/42 | 0.64 (0.44–0.94) | 0.022 |  |
|  | Insufficiently active and HighPNI | 78/11 | 0.29 (0.15–0.54) | <0.001 |  | 78/2 | 0.14 (0.03–0.59) | 0.007 |  | 78/9 | 0.36 (0.18–0.73) | 0.005 |  |
|  | Sufficiently active and LowPNI | 34/9 | 0.41 (0.20–0.82) | 0.012 |  | 34/2 | 0.32 (0.08–1.37) | 0.125 |  | 34/7 | 0.45 (0.20–1.01) | 0.054 |  |
|  | Insufficiently active and LowPNI | 74/16 | 0.47 (0.28–0.80) | 0.006 |  | 74/3 | 0.24 (0.07–0.79) | 0.019 |  | 74/13 | 0.62 (0.34–1.13) | 0.118 |  |
| obesity |  |  |  |  | 0.163 |  |  |  | 0.151 |  |  |  | 0.785 |
| No | Inactive and LowPNI | 420/193 | Reference |  |  | 420/52 | Reference |  |  | 420/141 | Reference |  |  |
|  | Sufficiently active and HighPNI | 85/8 | 0.30 (0.15–0.62) | 0.001 |  | 85/4 | 0.43 (0.15–1.21) | 0.108 |  | 85/4 | 0.23 (0.09–0.64) | 0.005 |  |
|  | Inactive and HighPNI | 388/98 | 0.80 (0.62–1.03) | 0.084 |  | 388/35 | 0.92 (0.59–1.43) | 0.714 |  | 388/63 | 0.75 (0.55–1.02) | 0.065 |  |
|  | Insufficiently active and HighPNI | 290/53 | 0.60 (0.44–0.82) | 0.002 |  | 290/22 | 0.79 (0.47–1.34) | 0.381 |  | 290/31 | 0.52 (0.35–0.78) | 0.002 |  |
|  | Sufficiently active and LowPNI | 98/17 | 0.38 (0.23–0.63) | <0.001 |  | 98/4 | 0.30 (0.11–0.85) | 0.024 |  | 98/13 | 0.42 (0.24–0.75) | 0.003 |  |
|  | Insufficiently active and LowPNI | 233/63 | 0.58 (0.43–0.78) | <0.001 |  | 233/19 | 0.61 (0.36–1.05) | 0.074 |  | 233/44 | 0.58 (0.41–0.83) | 0.003 |  |
| Yes | Inactive and LowPNI | 308/120 | Reference |  |  | 308/43 | Reference |  |  | 308/77 | Reference |  |  |
|  | Sufficiently active and HighPNI | 56/5 | 0.33 (0.13–0.81) | 0.016 |  | 56/3 | 0.45 (0.14–1.48) | 0.188 |  | 56/2 | 0.23 (0.06–0.93) | 0.04 |  |
|  | Inactive and HighPNI | 259/58 | 0.58 (0.42–0.80) | <0.001 |  | 259/21 | 0.55 (0.32–0.94) | 0.029 |  | 259/37 | 0.58 (0.39–0.87) | 0.008 |  |
|  | Insufficiently active and HighPNI | 128/9 | 0.26 (0.13–0.52) | <0.001 |  | 128/3 | 0.20 (0.06–0.65) | 0.007 |  | 128/6 | 0.29 (0.13–0.69) | 0.005 |  |
|  | Sufficiently active and LowPNI | 35/4 | 0.38 (0.14–1.06) | 0.064 |  | 35/2 | 0.37 (0.09–1.58) | 0.179 |  | 35/2 | 0.39 (0.09–1.64) | 0.199 |  |
|  | Insufficiently active and LowPNI | 120/22 | 0.55 (0.34–0.87) | 0.01 |  | 120/7 | 0.46 (0.20–1.03) | 0.059 |  | 120/15 | 0.60 (0.34–1.05) | 0.073 |  |
| CancerCategory |  |  |  |  | 0.409 |  |  |  | 0.302 |  |  |  | 0.597 |
| Non-obesity-related cancer | Inactive and LowPNI | 441/188 | Reference |  |  | 441/54 | Reference |  |  | 441/134 | Reference |  |  |
|  | Sufficiently active and HighPNI | 100/11 | 0.40 (0.21–0.74) | 0.003 |  | 100/6 | 0.71 (0.30–1.69) | 0.444 |  | 100/5 | 0.26 (0.11–0.65) | 0.004 |  |
|  | Inactive and HighPNI | 431/100 | 0.71 (0.56–0.92) | 0.008 |  | 431/36 | 0.83 (0.54–1.28) | 0.41 |  | 431/64 | 0.67 (0.49–0.91) | 0.01 |  |
|  | Insufficiently active and HighPNI | 322/42 | 0.50 (0.35–0.71) | <0.001 |  | 322/20 | 0.78 (0.46–1.35) | 0.376 |  | 322/22 | 0.39 (0.24–0.62) | <0.001 |  |
|  | Sufficiently active and LowPNI | 98/19 | 0.46 (0.28–0.74) | 0.001 |  | 98/6 | 0.52 (0.22–1.24) | 0.142 |  | 98/13 | 0.44 (0.24–0.78) | 0.005 |  |
|  | Insufficiently active and LowPNI | 237/62 | 0.60 (0.44–0.80) | <0.001 |  | 237/20 | 0.72 (0.42–1.22) | 0.219 |  | 237/42 | 0.56 (0.39–0.80) | 0.001 |  |
| Obesity-related cancer | Inactive and LowPNI | 287/125 | Reference |  |  | 287/41 | Reference |  |  | 287/84 | Reference |  |  |
|  | Sufficiently active and HighPNI | 41/2 | 0.13 (0.03–0.54) | 0.005 |  | 41/1 | 0.14 (0.02–1.02) | 0.053 |  | 41/1 | 0.12 (0.02–0.87) | 0.036 |  |
|  | Inactive and HighPNI | 216/56 | 0.71 (0.52–0.99) | 0.042 |  | 216/20 | 0.71 (0.41–1.23) | 0.225 |  | 216/36 | 0.70 (0.47–1.05) | 0.083 |  |
|  | Insufficiently active and HighPNI | 96/20 | 0.50 (0.31–0.82) | 0.006 |  | 96/5 | 0.33 (0.13–0.84) | 0.02 |  | 96/15 | 0.60 (0.34–1.06) | 0.076 |  |
|  | Sufficiently active and LowPNI | 35/2 | 0.14 (0.03–0.57) | 0.006 |  | 35/0 | NE |  |  | 35/2 | 0.23 (0.06–0.95) | 0.042 |  |
|  | Insufficiently active and LowPNI | 116/23 | 0.53 (0.34–0.84) | 0.007 |  | 116/6 | 0.39 (0.16–0.93) | 0.035 |  | 116/17 | 0.60 (0.35–1.03) | 0.065 |  |
| Cancer subtype |  |  |  |  | 0.666 |  |  |  | 0.549 |  |  |  | 0.563 |
| SkinNonMelanoma | Inactive and LowPNI | 110/49 | Reference |  |  | 110/10 | Reference |  |  | 110/39 | Reference |  |  |
|  | Sufficiently active and HighPNI | 23/1 | 0.19 (0.03–1.38) | 0.1 |  | 23/1 | 1.15 (0.13–10.02) | 0.898 |  | 23/0 | NE |  |  |
|  | Inactive and HighPNI | 103/23 | 0.52 (0.30–0.90) | 0.019 |  | 103/3 | 0.36 (0.08–1.49) | 0.158 |  | 103/20 | 0.59 (0.33–1.07) | 0.085 |  |
|  | Insufficiently active and HighPNI | 102/11 | 0.47 (0.23–0.96) | 0.037 |  | 102/4 | 0.86 (0.22–3.39) | 0.825 |  | 102/7 | 0.39 (0.16–0.93) | 0.035 |  |
|  | Sufficiently active and LowPNI | 29/2 | 0.21 (0.05–0.91) | 0.037 |  | 29/1 | 0.76 (0.08–6.82) | 0.808 |  | 29/1 | 0.13 (0.02–0.95) | 0.045 |  |
|  | Insufficiently active and LowPNI | 60/10 | 0.53 (0.26–1.08) | 0.082 |  | 60/5 | 1.02 (0.30–3.44) | 0.974 |  | 60/5 | 0.36 (0.14–0.94) | 0.037 |  |
| Breast | Inactive and LowPNI | 129/44 | Reference |  |  | 129/16 | Reference |  |  | 129/28 | Reference |  |  |
|  | Sufficiently active and HighPNI | 11/0 | NE |  |  | 11/0 | NE |  |  | 11/0 | NE |  |  |
|  | Inactive and HighPNI | 93/21 | 0.89 (0.50–1.56) | 0.68 |  | 93/9 | 1.07 (0.43–2.65) | 0.889 |  | 93/12 | 0.75 (0.36–1.58) | 0.449 |  |
|  | Insufficiently active and HighPNI | 56/11 | 0.82 (0.40–1.68) | 0.585 |  | 56/3 | 0.58 (0.15–2.21) | 0.427 |  | 56/8 | 0.86 (0.37–2.03) | 0.732 |  |
|  | Sufficiently active and LowPNI | 15/1 | 0.27 (0.03–2.06) | 0.206 |  | 15/0 | NE |  |  | 15/1 | 0.70 (0.09–5.46) | 0.734 |  |
|  | Insufficiently active and LowPNI | 61/11 | 1.08 (0.53–2.20) | 0.823 |  | 61/4 | 0.99 (0.31–3.15) | 0.987 |  | 61/7 | 0.99 (0.39–2.48) | 0.979 |  |
| Prostate | Inactive and LowPNI | 122/70 | Reference |  |  | 122/23 | Reference |  |  | 122/47 | Reference |  |  |
|  | Sufficiently active and HighPNI | 23/5 | 0.37 (0.14–0.97) | 0.042 |  | 23/3 | 0.80 (0.22–2.90) | 0.731 |  | 23/2 | 0.20 (0.05–0.88) | 0.033 |  |
|  | Inactive and HighPNI | 90/28 | 0.58 (0.36–0.92) | 0.022 |  | 90/13 | 0.75 (0.36–1.54) | 0.43 |  | 90/15 | 0.47 (0.25–0.88) | 0.018 |  |
|  | Insufficiently active and HighPNI | 48/9 | 0.34 (0.16–0.70) | 0.004 |  | 48/3 | 0.34 (0.09–1.21) | 0.094 |  | 48/6 | 0.34 (0.14–0.85) | 0.021 |  |
|  | Sufficiently active and LowPNI | 28/5 | 0.28 (0.11–0.73) | 0.009 |  | 28/2 | 0.41 (0.09–1.88) | 0.253 |  | 28/3 | 0.22 (0.06–0.76) | 0.016 |  |
|  | Insufficiently active and LowPNI | 73/21 | 0.34 (0.20–0.58) | <0.001 |  | 73/3 | 0.20 (0.06–0.70) | 0.012 |  | 73/18 | 0.35 (0.19–0.65) | <0.001 |  |
| Other | Inactive and LowPNI | 367/150 | Reference |  |  | 367/46 | Reference |  |  | 367/104 | Reference |  |  |
|  | Sufficiently active and HighPNI | 84/7 | 0.25 (0.12–0.54) | <0.001 |  | 84/3 | 0.29 (0.09–0.95) | 0.04 |  | 84/4 | 0.23 (0.08–0.63) | 0.004 |  |
|  | Inactive and HighPNI | 361/84 | 0.71 (0.54–0.93) | 0.015 |  | 361/31 | 0.81 (0.51–1.30) | 0.387 |  | 361/53 | 0.65 (0.47–0.92) | 0.014 |  |
|  | Insufficiently active and HighPNI | 212/31 | 0.47 (0.32–0.70) | <0.001 |  | 212/15 | 0.59 (0.32–1.08) | 0.088 |  | 212/16 | 0.40 (0.23–0.69) | <0.001 |  |
|  | Sufficiently active and LowPNI | 61/13 | 0.38 (0.21–0.67) | 0.001 |  | 61/3 | 0.29 (0.09–0.94) | 0.039 |  | 61/10 | 0.43 (0.22–0.84) | 0.014 |  |
|  | Insufficiently active and LowPNI | 159/43 | 0.56 (0.39–0.79) | 0.001 |  | 159/14 | 0.62 (0.33–1.14) | 0.125 |  | 159/29 | 0.53 (0.34–0.80) | 0.003 |  |

PNI: prognostic nutritional index; PA: physical activity; HR: hazard ratio; CI: confidence interval

N: the number of participants in each stratum; Events: the number of outcome events in that stratum. NE denotes not estimable due to sparse/zero events leading to monotone likelihood/non-convergence in Cox regression.

Model 3 was fully adjusted, including age, sex, race, marital status, education level, family poverty income ratio, smoking status, alcohol consumption, PA, BMI, hypertension, hyperlipidemia, diabetes, obesity, abdominal obesity and cancer subtype.

Low: PNI≤51.0; High: PNI>51.0

Inactive: 0 MET-min/week, Insufficiently active: 1-599 MET-min/week, Sufficiently active: ≥600 MET-min/week

*P*-value<0.05 was considered significant.

**Table S5. Sensitivity analysis using PNI tertiles in Model 3.**

| Outcome | N/Events | HR (95% CI) | *P* value |
| --- | --- | --- | --- |
| All-cause |  |  |  |
| T1 | 897/347 | 1.00 (ref) |  |
| T2 | 785/184 | 0.71 (0.59-0.85) | <0.001 |
| T3 | 742/119 | 0.58 (0.47-0.72) | <0.001 |
| CVD |  |  |  |
| T1 | 893/93 | 1.00 (ref) |  |
| T2 | 785/45 | 0.64 (0.45-0.92) | 0.017 |
| T3 | 742/29 | 0.58 (0.38-0.89) | 0.012 |
| Cancer |  |  |  |
| T1 | 893/110 | 1.00 (ref) |  |
| T2 | 785/58 | 0.66 (0.48-0.91) | 0.011 |
| T3 | 742/47 | 0.63 (0.44-0.90) | 0.01 |
| Non-cancer |  |  |  |
| T1 | 893/237 | 1.00 (ref) |  |
| T2 | 785/126 | 0.74 (0.59-0.92) | 0.006 |
| T3 | 742/72 | 0.55 (0.42-0.72) | <0.001 |

PNI: prognostic nutritional index; CVD mortality, cardiovascular disease mortality; HR, hazard ratio; CI, confidence interval

N: the number of participants in each stratum; Events: the number of outcome events in that stratum.

PNI tertiles were defined using cutpoints of 49.5 and 53.5 (T1: ≤49.5; T2: 49.5-53.5; T3: >53.5).

Model 3, including age, sex, race, marital status, education level, family poverty income ratio, smoking status, alcohol consumption, PA, BMI, hypertension, hyperlipidemia, diabetes, obesity, abdominal obesity and cancer subtype.

P values < 0.05 indicate evidence against proportional hazards.

**Table S6. Sensitivity analysis using PNI dichotomized at the median (cutpoint = 51) in Model 3.**

| Outcome | N/Events | HR (95% CI) | *P* value |
| --- | --- | --- | --- |
| All-cause mortality |  |  |  |
| Low | 1206/231 | 1.00 (ref) |  |
| High | 1214/419 | 0.73 (0.62-0.86) | <0.001 |
| CVD mortality |  |  |  |
| Low | 1206/51 | 1.00 (ref) |  |
| High | 1214/116 | 0.64 (0.45-0.89) | 0.008 |
| Cancer mortality |  |  |  |
| Low | 1206/88 | 1.00 (ref) |  |
| High | 1214/127 | 0.83 (0.62-1.09) | 0.179 |
| Non-cancer mortality |  |  |  |
| Low | 1206/143 | 1.00 (ref) |  |
| High | 1214/292 | 0.67 (0.55-0.83) | <0.001 |

PNI: prognostic nutritional index; CVD mortality, cardiovascular disease mortality; HR, hazard ratio; CI, confidence interval

N: the number of participants in each stratum; Events: the number of outcome events in that stratum.

PNI categorized as low (PNI ≤ 51) or high (PNI > 51).

Model 3, including age, sex, race, marital status, education level, family poverty income ratio, smoking status, alcohol consumption, PA, BMI, hypertension, hyperlipidemia, diabetes, obesity, abdominal obesity and cancer subtype.

P values < 0.05 indicate evidence against proportional hazards.

**Table S7. Proportional hazards (PH) assumption assessment for Model 3 using Schoenfeld residual tests (key terms).**

| Outcome | Term | χ² | df | P value |
| --- | --- | --- | --- | --- |
| All-cause mortality | PNI | 18.98 | 1 | <0.001 |
|  | PA | 5.95 | 2 | 0.0509 |
|  | age | 10.7 | 1 | 0.0011 |
|  | GLOBAL | 58.63 | 20 | <0.001 |
| Non-cancer mortality | PNI | 11.74 | 1 | <0.001 |
|  | PA | 5.97 | 2 | 0.0506 |
|  | age | 5.31 | 1 | 0.0212 |
|  | GLOBAL | 35.6 | 20 | 0.0171 |
| Cancer mortality | PNI | 8.45 | 1 | 0.0037 |
|  | PA | 0.68 | 2 | 0.712 |
|  | age | 2.14 | 1 | 0.1437 |
|  | GLOBAL | 36.52 | 20 | 0.0134 |
| CVD mortality | PNI | 5.11 | 1 | 0.0238 |
|  | PA | 1.66 | 2 | 0.4352 |
|  | age | 4.99 | 1 | 0.0254 |
|  | GLOBAL | 29.57 | 20 | 0.077 |

PNI: prognostic nutritional index; CVD mortality, cardiovascular disease mortality; PA: physical activity; df: degrees of freedom

P values < 0.05 indicate evidence against proportional hazards.

**Table S8. Proportional hazards (PH) assumption assessment for Model 3 (all covariates).**

|  | All-cause mortality | | | Cancer mortality | | | Non-cancer mortality | | | CVD mortality | | |
| --- | --- | --- | --- | --- | --- | --- | --- | --- | --- | --- | --- | --- |
| Term | χ² | df | P value | χ² | df | P value | χ² | df | P value | χ² | df | P value |
| GLOBAL | 58.63 | 20 | <0.001 | 36.52 | 20 | 0.0134 | 35.6 | 20 | 0.0171 | 29.57 | 20 | 0.077 |
| BMI | 0.42 | 1 | 0.5178 | 1.23 | 1 | 0.2682 | 0.02 | 1 | 0.8924 | 0.03 | 1 | 0.8527 |
| PA | 5.95 | 2 | 0.0509 | 0.68 | 2 | 0.712 | 5.97 | 2 | 0.0506 | 1.66 | 2 | 0.4352 |
| PIR | 0 | 1 | 0.9761 | 0.47 | 1 | 0.4942 | 0.07 | 1 | 0.792 | 0.11 | 1 | 0.7383 |
| PNI | 18.98 | 1 | <0.001 | 8.45 | 1 | 0.0037 | 11.74 | 1 | <0.001 | 5.11 | 1 | 0.0238 |
| Smoke | 0.63 | 2 | 0.7286 | 1.78 | 2 | 0.4113 | 0.1 | 2 | 0.9535 | 4.83 | 2 | 0.0894 |
| age | 10.7 | 1 | 0.0011 | 2.14 | 1 | 0.1437 | 5.31 | 1 | 0.0212 | 4.99 | 1 | 0.0254 |
| diabetes | 0.28 | 1 | 0.5998 | 0.85 | 1 | 0.356 | 0.02 | 1 | 0.8943 | 0.08 | 1 | 0.7722 |
| drinking | 0.13 | 1 | 0.7154 | 1.57 | 1 | 0.2105 | 0.13 | 1 | 0.7136 | 0.38 | 1 | 0.5397 |
| dyslipidemia | 0.05 | 1 | 0.818 | 1 | 1 | 0.3161 | 0.07 | 1 | 0.7951 | 0.04 | 1 | 0.8403 |
| education | 0.58 | 2 | 0.7492 | 2.51 | 2 | 0.2853 | 0.08 | 2 | 0.9604 | 3.74 | 2 | 0.1544 |
| hypertension | 1.56 | 1 | 0.2122 | 0.37 | 1 | 0.5406 | 1.08 | 1 | 0.2986 | 0.1 | 1 | 0.757 |
| marital | 1.61 | 1 | 0.2048 | 0.2 | 1 | 0.6552 | 3.37 | 1 | 0.0665 | 0 | 1 | 0.9775 |
| obesity | 0 | 1 | 0.9966 | 0.12 | 1 | 0.7274 | 0.03 | 1 | 0.8551 | 0.87 | 1 | 0.3515 |
| race | 6.23 | 3 | 0.1007 | 8.39 | 3 | 0.0385 | 1.62 | 3 | 0.6547 | 6.35 | 3 | 0.0956 |
| sex | 0.1 | 1 | 0.7496 | 0 | 1 | 0.9622 | 0.32 | 1 | 0.5699 | 1.18 | 1 | 0.2768 |

PNI: prognostic nutritional index; CVD mortality, cardiovascular disease mortality; PA: physical activity; PIR: poverty income ratio; df: degrees of freedom

*P*-value<0.05 was considered significant.

**Table S9. Sensitivity analysis using an extended Cox model with time-varying coefficients for PNI and age (Model 3)**

| Outcome | Term | HR (95% CI) | P value |
| --- | --- | --- | --- |
| All-cause mortality | PA: Inactive | 1.00 (ref) |  |
|  | PA: Insufficiently active | 0.64 (0.52–0.77) | <0.001 |
|  | PA: Sufficiently active | 0.39 (0.28–0.56) | <0.001 |
|  | PNI | 0.76 (0.70–0.82) | <0.001 |
|  | PNI × log(time) | 1.06 (1.04–1.08) | <0.001 |
|  | Age × log(time) | 1.02 (1.01–1.04) | <0.001 |
| CVD mortality | PA: Inactive | 1.00 (ref) |  |
|  | PA: Insufficiently active | 0.79 (0.55–1.15) | 0.223 |
|  | PA: Sufficiently active | 0.37 (0.18–0.78) | 0.009 |
|  | PNI | 0.80 (0.69–0.92) | 0.002 |
|  | PNI × log(time) | 1.04 (1.00–1.08) | 0.039 |
|  | Age × log(time) | 1.04 (1.02–1.06) | <0.001 |
| Cancer mortality | PA: Inactive | 1.00 (ref) |  |
|  | PA: Insufficiently active | 0.64 (0.46–0.90) | 0.009 |
|  | PA: Sufficiently active | 0.43 (0.24–0.77) | 0.005 |
|  | PNI | 0.74 (0.66–0.84) | <0.001 |
|  | PNI × log(time) | 1.07 (1.03–1.10) | <0.001 |
|  | Age × log(time) | 1.02 (1.00–1.03) | 0.041 |
| Non-cancer mortality | PA: Inactive | 1.00 (ref) |  |
|  | PA: Insufficiently active | 0.64 (0.50–0.81) | <0.001 |
|  | PA: Sufficiently active | 0.38 (0.24–0.59) | <0.001 |
|  | PNI | 0.77 (0.70–0.85) | <0.001 |
|  | PNI × log(time) | 1.05 (1.03–1.08) | <0.001 |
|  | Age × log(time) | 1.03 (1.01–1.04) | <0.001 |

PNI: prognostic nutritional index; PA: physical activity; HR: hazard ratio; CI: confidence interval

Inactive: 0 MET-min/week. Insufficiently active: 1-599 MET-min/week,. Sufficiently active: ≥600 MET-min/week

*P*-value<0.05 was considered significant.
